# Understanding the intricacy of canid social systems: Structure and temporal stability of red fox (*Vulpes vulpes*) groups

**DOI:** 10.1371/journal.pone.0220792

**Published:** 2019-09-11

**Authors:** Jo Dorning, Stephen Harris

**Affiliations:** School of Biological Sciences, University of Bristol, Bristol, England, United Kingdom; University of Missouri Columbia, UNITED STATES

## Abstract

Red foxes have a highly flexible social system. Despite numerous studies worldwide, our understanding of the pattern and stability of fox social relationships remains limited. We applied social network analysis to camera trap data collected at high-quality foraging patches to examine the social structure of a population of urban red foxes. Foxes encountered a conspecific on 13% of patch visits, and had significant preferred and avoided companionships in all seasons. They also associated in communities that matched territorial space use, confirming that territories can be analysed separately to increase power without excluding too many social partners. Foxes maintained stable, long-term relationships with other territory residents, but the average longevity of relationships varied seasonally, suggesting that social connectivity, particularly between foxes from different social groups, is influenced by their annual life cycle. The probability of re-association after a given time lag was highest in spring and summer, during cub birth and rearing, and lowest in the winter mating season, when mean relationship duration was shorter. 33% of fox relationships lasted for four consecutive seasons and were probably between territory residents. 14% lasted for around 20 days and were probably between residents and visitors from adjacent territories. The majority (53%) lasted less than one day, particularly during dispersal and mating, and were probably between foxes from non-adjacent social groups. Social structure varied between groups; in one group the death of the dominant male caused significant social disruption for two seasons. This is the first application of social network analysis to multiple red fox social groups. However, our analyses were based on interactions at high quality food patches; social connections may differ when foxes are resting, travelling and foraging elsewhere in their territory. Our results will inform management practices, particularly for disease spread and population control.

## Introduction

Social structure affects a wide range of ecological, evolutionary and population processes [1–12], and social networks can link individual relationships to group and/or population-level processes [13]. Social differentiation, for instance, describes the extent to which association rate (the propensity for a pair of individuals to be encountered together) varies between dyads [14], and gregariousness defines an individual’s tendency to form associations [15]. Heterogeneity in association rate can be attributed to demographic effects such as birth, death and dispersal, or to preferred and avoided companionships [14]. Socially heterogeneous populations can be divided into clusters of individuals (communities) that associate more strongly with each other than with the rest of the population [16]. The composition of communities can help explain preferential associations, and community size indicates an individual’s number of potential associates over a period of time [14,17]. In some colonial [18] and fission-fusion [19,20] species, community membership is explained by overlapping space use, which may also play a critical role in defining social units for territorial species [21]. While communities may be comparable to social groups delineated by behavioural or spatial observations (territories), they can reveal more subtle substructures, particularly inter-group social links [22].

Temporal patterning of associations is a key feature of social structure [23], and comparing data from the same population in different seasons can reveal underlying effects of reproduction [24–28], food availability [29–31], parasitism [32] or environmental conditions [33–35]. When data are collected over sufficient timespans, lagged association rates (LAR) can be used to determine the temporal stability of relationships [36] and structural aspects of social organisation [14]. Understanding the social system of species of economic and conservation concern is particularly important [37–40], since this influences processes such as disease transmission [24,28,41] and resilience to perturbation [42–44].

The red fox (*Vulpes vulpes*) is the most widespread terrestrial mammal [45] and globally important as an invasive species, predator, competitor and vector of various diseases. While plasticity in social organisation is a key factor in the species’ success [46–49], fox social groups are difficult to define [50] and the only network analysis thus far involved just four animals [51]. Since red foxes are solitary foragers with apparently low contact rates [52], have a low average life expectancy [53], commonly disperse in their first year [54], and exhibit significant seasonal variation in ranging patterns and social behaviour [52,55–57], relationships may not be long-lasting, particularly in heavily culled populations [58]. While red foxes form spatiotemporally stable social groups in many British cities [47,49,59,60], frequent intergroup movements and foraging on adjacent territories, particularly by subordinates [61], make it difficult to determine social group membership [50].

To further our understanding of red fox social organisation and population dynamics, we used social network analyses of a high-density urban fox population to determine (1) whether red foxes associate in distinct communities (i.e. individuals meet and interact with foxes from other social groups), (2) whether these communities can be explained by territorial space use, and (3) whether foxes maintain long-term social relationships with other group members. These data are needed to advance our understanding of fox social behaviour and inform population management programmes.

## Materials and methods

### Study area and data collection

The study was conducted in an urban area of approximately 1.5 km^2^ in the northwest suburbs of Bristol, UK. The habitat consists predominantly of 1930s semi-detached housing with medium-sized gardens and had one of the highest fox densities in the city [62]. It is the site of an intensive study covering four decades and there is a long-term record of population density and social group structure based on radio-tracking and capture-mark-recapture data [48,49,56,63,64].

Between July 2013 and June 2015 we positioned camera traps in 4 to 6 residential back gardens in each of seven fox territories; cameras were positioned to record visits to locations (food patches) where the householders provisioned the foxes regularly [61,65]. All residents at our field sites gave us permission to conduct the study in their gardens. The cameras were continuously active for 40 days in each of four consecutive seasons: spring (March-May; birth and early cub-rearing), summer (June-August; late cub rearing, onset of juvenile independence), autumn (September-November; onset of dispersal) and winter (December-February; peak dispersal and mating). Consecutive surveys in the same territory were separated by a minimum of 39 days, so each seasonal survey was considered independent, and a finite number of foxes could be captured (i.e. photographed) in each survey. Not all territories were surveyed concurrently due to logistical constraints. Full details of the timing of the surveys in each territory, camera trapping techniques, data collection and handling, are given in [61,65]. We only included foxes > 5 months old in the analyses and identified the individual fox in 99% of capture records; full details on the techniques used to identify each fox, and levels of accuracy, are given in [65].

### Data preparation and network construction

Population-level analyses were conducted on a single dataset containing association data from all territories and seasons. Territory-level analyses were conducted on 28 separate networks for each territory and season because foxes show clear seasonal variations in behaviour which may affect social networks e.g. [52,55,57]. Some foxes visited several territories so appeared in more than one interconnected network of animals behaving socially [50,61,65]. To avoid preconceived assumptions about social structure, there was no criterion for inclusion or exclusion of an individual from a particular network, and so all foxes were retained and territories analysed independently. For the same reason, seasonal networks were compared within rather than between territories, as sampling effort (the number and spacing of camera sites) varied slightly between territories.

Since network data collected by different methods are not directly comparable [66,67], we used data from a similar number of patches in each territory and season (four patches per season in territories 1–4, 6 and 7, five patches per season in territory 5; in territories 1–6 the same patches were used in every season) to facilitate network comparisons [21].

Spatiotemporal associations at foraging patches were inferred using the ‘gambit of the group’, i.e. each animal in a group was assumed to be associating with every other individual in that group [23] and social networks constructed in SOCPROG v.2.6 [68]. Data were input in dyadic format, so if an individual associated with more than one conspecific during a patch visit, each dyadic association was recorded on a separate row. Foxes are primarily active between 20:00–04:00 [55] so sampling periods were days starting and ending at noon, a natural break in activity to ensure independent sampling [69].

We used the simple ratio index (SRI) to estimate the proportion of time each dyad spent associated, scaled between 0 (never observed together) and 1 (always observed together). The SRI is statistically unbiased and is recommended if associations are accurate and symmetric, with all identified associates and individuals equally likely to be identified whether associated or alone [14,70,71]. All assumptions were met by this dataset. The SRI was calculated by:-

$${SRI}_{AB}=\frac{x}{x+Y_{AB}+Y_{A}+Y_{B}}$$

where x is the number of sampling periods in which individuals A and B were associated, Y_AB_ is the number of sampling periods in which A and B were identified but not associated, and Y_A_ and Y_B_ the number of sampling periods in which only A or only B was identified [71].

The SRI was used to estimate the proportion of time each dyad spent associated, scaled between 0 (never observed together) and 1 (always observed together). In social network analyses it is common to apply an observation threshold of 2–6 sightings [14,69] to reduce bias from poorly-studied individuals [14]. We excluded foxes observed on < 5 days because this marked a discontinuity in the distribution of individual sighting frequencies (Fig A in S1 Appendix). Plotting the effect of an increasing sighting threshold on social differentiation and power also showed a clear change in line trajectory at a minimum observation of 5 days. Some foxes, referred to hereafter as isolates, were always observed alone so were not inter-connected to the main network component.

### Social differentiation and power

We used the coefficient of variation of the association indices (*S*) to quantify social differentiation, where *S* < 0.2 indicates a poorly differentiated society and *S* > 0.8 a strongly differentiated society [13,14,72]. To illustrate the level of social differentiation, we plotted frequency distributions of dyadic SRIs and individual mean and maximum SRIs as proportions. The mean SRI indicates the proportion of time an individual spends associating with conspecifics and the maximum SRI represents the proportion of time associating that is spent with a particular individual, i.e. the individual’s closest companion. We constructed network diagrams in NetDraw [73], with strongly-bonded dyads positioned closer together by spring-embedding, to display the strength and patterning of relationships in a more easily-interpreted format. We used the Pearson’s correlation coefficient (*r*) between the true and estimated association indices to assess the power of the dataset to describe the true social system, where a value of *r* = 1 indicated a perfect representation of social structure and *r* = 0.4 a moderate representation [14,72]. Both measures were estimated by maximum likelihood and standard errors calculated from 100 bootstrap replicates.

### Preferred and avoided companionships

To determine whether associations were active social groupings or simply random aggregations at shared resource patches, we used the modified Manly/Bejder permutation procedure in SOCPROG [68,69,74] to test the null hypothesis that dyads had no preferred or avoided social partners within or between sampling periods (days). As not all foxes were seen every day, we randomised groups within days to account for individual differences in detectability and the non-independence of associations recorded in the same day [75]. This permutation method holds constant the number of groups each individual was observed in each day, and the size of those groups, but does not control for individual differences in gregariousness, i.e. their tendency to form associations [15]. However, isolates were excluded since this method is sensitive to their inclusion. Long-term (between-day) preferred or avoided companionships were indicated by a higher coefficient of variation (CV) of SRIs in the observed data than in 95% of the permuted datasets. Short-term (within-day) preferred companions were indicated by a significantly lower mean in the observed data compared to 95% of the permuted datasets. This also tests for individual differences in gregariousness, indicated by a higher standard deviation (of the mean number of foxes encountered per day) in the observed network compared to 95% of the permuted datasets. We also tested for non-random association by permuting associations (rather than groups) within days to control for gregariousness. This permutation method can only detect long-term preferred and avoided companionships and requires considerably more data [14] but controlling for gregariousness removes the possibility that the null hypothesis of random association will be rejected when individuals differ in their tendency to associate but have no preference for particular associates (type 1 error) [14]. Both tests were run using 5000 permutations with 1000 trials per permutation, as pilot runs indicated 5000 permutations were enough to stabilise *p*-values.

### Community detection

To determine whether territories were responsible for fox social structure, we tested whether the population could be divided into communities that overlapped territories. We used SOCPROG to implement two methods of community detection: eigenvector-based (non-hierarchical) community detection [76] and average-linkage hierarchical cluster analysis [77]. Both methods control for individual differences in gregariousness and subdivide the population into communities until modularity (Q), defined as the difference between the proportion of total associations observed within communities and the expected proportion if foxes associated randomly, is maximised. Q > 0.3 indicates a useful division [78]. When Q < 0.3 we assumed that all individuals belonged to the same community. The hierarchical method assumes that communities are nested within one another [23], whereas the eigenvector method optimises modularity over all possible divisions [76].

We used a Mantel test with 5000 permutations, each with 1000 trials, to check that associations were stronger within than between communities, where a significant difference was indicated by a positive *t*-value, a high *p-*value, and a positive matrix correlation. Community structure was plotted as a network diagram in NetDraw [73], with nodes (individuals) and weighted edges (SRI) arranged using spring-embedding from random start positions.

We determined whether foxes were consistently assigned to the same community over time by dividing the data into seasons and years to create eight datasets between summer 2013 and spring 2015. We excluded individuals observed on < 5 days in each season-year combination and used the two methods of community detection described above to divide each dataset into communities. We selected communities assigned by the method with the highest maximum modularity and calculated the proportion of individuals assigned to the same community across multiple seasons.

To verify that the space use of individuals in communities matched territory location, we counted the number of observations of individuals in each community that were recorded in each territory and used these to construct spatial profile histograms [18]. We calculated the percentage of visits individuals made to different patches and combined these to plot the percentage patch use for each community, with patches grouped by territory.

To quantify whether relationships based on spatiotemporal associations were comparable to relationships based on overlapping space use without requiring physical encounters, we redefined associations as ‘individuals observed in the same patch on the same day, but not necessarily at the same time’ and compared the spatiotemporal and spatial association matrices using a Dietz-*R* Mantel test [79]. We also ran the eigenvector community detection algorithm again to detect communities based on same-day patch use and compared these visually with communities based on associations.

### Temporal stability of relationships

We determined whether territories contained stable social groups rather than short-term clusters of individuals by calculating LARs for the whole dataset combined and separately for each territory, community and season. LARs represent the probability that a dyad will associate again after a given time lag [36]. Each LAR was compared to the null association rate (NAR), which is the association rate expected when foxes associate randomly [68]. If the LAR > NAR, this demonstrates the presence of non-random associations. There were no restrictions on the dataset as poorly sampled individuals have little impact on LAR estimates [14]. To describe the temporal pattern of change in each LAR, we fitted a set of exponential decay models [36] that approximated features of different social structures including preferred companions (permanent relationships lasting until death), casual acquaintances (temporary short- or long-term associations lasting days, months or years) and rapid disassociations (short-lived associations lasting less than a day). Initial starting parameters were 0.5 for all models; these were adjusted and the models refitted if standard errors were large. The best-fitting model was indicated by the lowest QAIC; we report the top two models if ΔQAIC < 2. We determined the precision of the LAR and model parameters using the temporal jack-knife procedure, where days were omitted in turn [36].

To aid interpretation of the LAR, we also calculated the lagged identification rate (LIR) for the whole dataset combined, with no restrictions. LIR is a non-social measure that represents the probability that a fox identified in the study area is the same as a randomly chosen fox some time lag later [80]. A decline in LIR indicates that animals are leaving the study area through emigration or mortality, so corresponding drops in both LAR and LIR suggest that changes in association patterns are explained by demography rather than social dissociation [81]. We fitted exponential models to assess the time periods over which foxes were identified in the study area [68]. The best-fitting model had the lowest QAIC.

We used Mantel permutation tests to examine whether patterns of associations in territories were stable across seasons, by testing for correlations between pairs of matched SRI matrices from different seasons in the same territory. Matched matrices only contained individuals observed in both seasons. We ran both the standard Mantel *z*-test [82] and the Dietz *R*-test [79], which is less sensitive to extreme values, three times using 10,000 permutations and calculated the mean matrix correlation and *p*-value, where *p* < 0.05 indicates a significant correlation between matrices. Test results were grouped by the time difference between the compared seasons in the order of data collection, where 1 indicates consecutive seasons, 2 indicates a gap of 1 season and 3 a gap of two seasons. Although results from both Mantel and Dietz *R*-tests are reported, *p*-values from the more robust Dietz’s *R* were used to determine the overall consistency of association patterns between seasons: we combined *p*-values with time differences of 1 season (2 tests, between two independent pairs of seasons) or 2 seasons (2 tests) within territories using Fisher’s Omnibus Test [83] in R version 3.2.4 [84] using the package *metap* (version 0.60) [85].

## Results

Survey effort is summarized in Table A in S2 Appendix. Of the 175 foxes > 5 months old that we identified, 174 visited the patches used in the dataset standardised for network analysis: 83 were seen on ≥ 5 days across the entire study period. We recorded 38,273 observations of these 83 individuals, of which 3914 were true associations (not self-associations). After removing individuals observed on < 5 days per survey, this reduced to 34,313, of which 3909 were true associations. When associations with multiple individuals were pooled within patch visits, foxes encountered at least one other individual during 13% of patch visits i.e. they were alone on 87% of visits. On average we observed 139.6 true associations per survey (SD = 102.0): true associations were most common in autumn (mean/territory ± SD = 173.7 ± 117.1) and in territory 1 (mean/survey = 280.8 ± 64.3), and least common in winter (mean/territory = 97.3 ± 65.2) and in territory 2 (mean/survey = 68.5 ± 48.4). In each territory we observed a mean of 6.5 ± 3.0 individuals per day and 8.6 ± 4.6 per survey, excluding foxes seen on < 5 days. The mean number of individuals recorded per survey was highest in winter (*N* = 10.7 ± 5.5) and lowest in summer (*N* = 6.6 ± 3.0), highest in territory 6 (*N* = 15.0 ± 7.4) and lowest in territory 7 (*N* = 4.5 ± 0.6).

### Social differentiation and power

Social differentiation was high in the combined dataset (*S* ± SE = 1.255 ± 0.012), indicating that association patterns were highly variable, but the correlation coefficient between true and estimated SRIs was low (*r* ± SE = 0.197 ± 0.003), suggesting limited power to detect the true social system. This is probably due to combining data from territories that rarely comingled, leading to high social differentiation but a low mean number of associations per dyad (mean = 0.93, Table A in S2 Appendix). Power was higher when calculated separately for each territory and season: *r* > 0.4 for 25 of the 28 networks and the 3 networks with low *r* also had low mean numbers of associations per dyad. Power was positively correlated with the mean number of associations, supporting the need for more data in some cases. Networks with *r* < 0.4 have limited power to detect the true social system and should be interpreted with caution. Overall, there was greater power to detect the true social system when the data were divided by territory and season than when pooled.

In networks separated by territory and season, some dyads were strongly associated but the majority had an association index of zero, suggesting that they never associated during the study period (Fig B in S1 Appendix). Non-zero association indices were least common in winter, but there was high between-territory variation. Territory 7 had a high proportion of strong relationships in all seasons, and territory 6 had a consistently high proportion of non-associating dyads, possibly because large networks may contain more non-residents that are less well-integrated into the social unit. Individual mean and maximum SRIs (Fig C in S1 Appendix) showed that some individuals in each network spent little or no time being social, while others spent up to 65% of their time being social (see territory 7 in spring) and up to 100% of that time with a single preferred companion (see territory 3 in spring).

### Preferred and avoided companionships

The Manly/Bejder test on the pooled dataset (groups permuted within days and all isolates excluded) showed that foxes have preferred short- (mean_*obs*_ = 0.008, mean_*rand*_ = 0.012, *p* < 0.001) and long-term (CV_*obs*_ = 5.565, CV_*rand*_ = 4.033, *p* < 0.001) companions, with significant individual differences in gregariousness (SD_*obs*_ = 0.163, SD_*rand*_ = 0.129, *p* < 0.001). Foxes still associated non-randomly when isolates were included and associations permuted within days to control for gregariousness (CV_*obs*_ = 6.355, CV_*rand*_ = 4.625, *p* < 0.001).

When the data were separated by territory and season, two networks in spring and two in summer contained too few associations to permute associations within days (Table B in S2 Appendix), but combined *p*-values from the remaining networks confirmed that associations were still non-random in all seasons (Table 1). All networks could be randomised when groups were permuted within days and isolates excluded. There were significant long-term companionships in 22/28 networks at the 0.05 level of significance and 24/28 networks at the 0.1 level (Table B in S2 Appendix). Significant short-term (within-day) companionships were less common and confirmed in just 7/28 networks at the 0.05 level of significance and 9/28 networks at the 0.1 level. To maximise sample size, networks were considered non-random if they contained significant long-term associations at the 0.1 level. Individual differences in gregariousness were detected in 13/28 networks at the 0.05 level of significance and 15/28 networks at the 0.1 level (Table B in S2 Appendix). Combined *p*-values from all territories confirmed that all measures were significant in all seasons at the 0.05 level, with the exception of summer, when foxes had no detectable preferences for short-term companionships (Table 1).

**Table 1:** *P-*values combined using Fisher’s omnibus test, from the Manly/Bejder test for preferred and avoided companions using two permutation methods^a^^,^^b^.

	Long-term (CV)^a^			Short-term (mean)^b^			Long-term (CV)^b^			Gregariousness (SD)^b^		
Season	χ^2^	df	*p*	χ^2^	df	*p*	χ^2^	df	*p*	χ^2^	df	*p*
Spring	35.163	10	**<0.001**	30.339	14	**0.007**	70.032	14	**<0.001**	44.113	14	**<0.001**
Summer	47.587	10	**<0.001**	20.177	14	0.125	64.123	14	**<0.001**	64.894	14	**<0.001**
Autumn	58.476	14	**<0.001**	27.109	14	**0.019**	82.958	14	**<0.001**	73.796	14	**<0.001**
Winter	51.348	14	**<0.001**	38.138	14	**<0.001**	64.354	14	**<0.001**	37.369	14	**<0.001**

Bold values indicate significance at *p* < 0.05.

^a^ Associations were permuted within sampling periods (days) and isolates were included;

^b^ groups were permuted within days and isolates were excluded.

### Social structure of the population

Eigenvector community detection divided the population into 10 communities, and high modularity (Q = 0.834) indicated an accurate division. Average-linkage clustering detected 34 communities, many of which were isolates, and had a lower modularity (Q = 0.822), so we report communities from the eigenvector method. Associations were significantly higher within than between communities (Mantel test: matrix correlation = 0.436, *t* = 21.49, *p* = 1.000). Communities 1–7 contained 4–16 adults (Table 2), but community 8 contained just two foxes and the relatively low number of observations of this pair suggested that they were part of a community not included in this study. Individuals in communities 9 and 10 were all isolates and could not be assigned to a community. Communities contained a roughly equal sex ratio and many more subordinates than dominants, of which there was usually one of each sex. Territories contained substantially more foxes than communities (Table 3) and a male-biased sex ratio, mainly due to the high number of subordinates.

**Table 2:** Communities delineated by eigenvector community detection based on data from the whole study period (modularity = 0.834) and the number of observations of individuals in each community that were recorded in each territory.

Community	*N*	M Dom	F Dom	M Sub	F Sub	M Ust	F Ust	Usx Ust	Number of observations per territory						
									T1	T2	T3	T4	T5	T6	T7
**C1**	12	1	1	6	4	-	-	-	6437	29	5	-	-	-	-
**C2**	5	1	1	1	2	-	-	-	-	4764	138	-	-	-	-
**C3**	8	1	2	2	2	1	-	-	-	399	5127	-	-	-	-
**C4**	11	1	1	5	4	-	-	-	-	-	-	5131	117	1198	-
**C5**	16	1	1	11	3	-	-	-	-	-	-	100	5363	42	1
**C6**	13	2	1	5	5	-	-	-	-	-	-	1	128	4091	-
**C7**	4	1	1	-	2	-	-	-	-	-	-	-	-	-	4101
**C8**	2	1	1	-	-	-	-	-	-	-	-	-	9	108	42
C9^a^	11	1	-	7	1	1	-	1	941	3	13	-	19	61	2
C10^a^	1	-	-	1	-	-	-	-	-	-	-	-	1	5	-

Bold communities indicate those with high-confidence membership. Observations include both true- and self-associations. M = male, F = female, Usx = unknown sex, Dom = dominant, Sub = subordinate, Ust = unknown social status.

^a^ Membership of communities 9 and 10 was uncertain as all individuals were isolates.

**Table 3:** Membership of communities delineated in each season and year separately (season-year communities).

Year	Season	Community	Colour	*N*	M Dom	F Dom	M Sub	F Sub	M Ust
2013	Summer	C1	Yellow	7	1	1	1	4	-
2013	Autumn	C1	Yellow	10	1	1	6	2	-
		C2	Cyan	4	1	1	-	2	-
		C3	Red	5	1	1	2	1	-
		C4	Light blue	6	1	1	2	2	-
			White	1	-	-	-	1	-
2013–14	Winter	C1	Yellow	8	1	1	4	2	-
		C2	Cyan	5	1	1	1	1	1
		C3	Red	6	1	1	1	2	1
		C4	Light blue	6	1	1	3	1	-
		C5	Navy	10	1	1	6	2	-
			Green	5	1	-	2	2	-
2014	Spring	C1	Yellow	7	1	1	5	-	-
		C2	Cyan	4	1	1	-	2	-
		C3	Red	6	1	2	2	1	-
		C4	Light blue	4	1	1	1	1	-
		C5	Navy	5	1	1	2	1	-
			Green	2	-	-	1	1	-
			Pink	2	-	-	-	2	-
			Black	2	1	-	-	1	-
2014	Summer	C2	Cyan	4	1	1	-	2	-
		C3	Red	5	1	1	2	1	-
		C4	Light blue	4	1	1	1	1	-
		C5	Navy	4	1	1	1	1	-
		C6	Orange	8	1	1	3	3	-
		C7	Purple	4	1	2	-	1	-
2014	Autumn	C5	Navy	9	1	1	5	2	-
		C6	Orange	13	3	3	2	5	-
		C7	Purple	4	1	1	-	2	-
2014–15	Winter	C4	Light blue	5	1	1	1	2	-
		C6	Orange	6	-	1	2	3	-
		C7	Purple	4	1	1	-	2	-
2015	Spring	C4	Light blue	2	1	1	-	-	-
		C6	Orange	4	1	1	1	1	-
		C7	Purple	4	1	1	-	2	-

**Fig 1:**
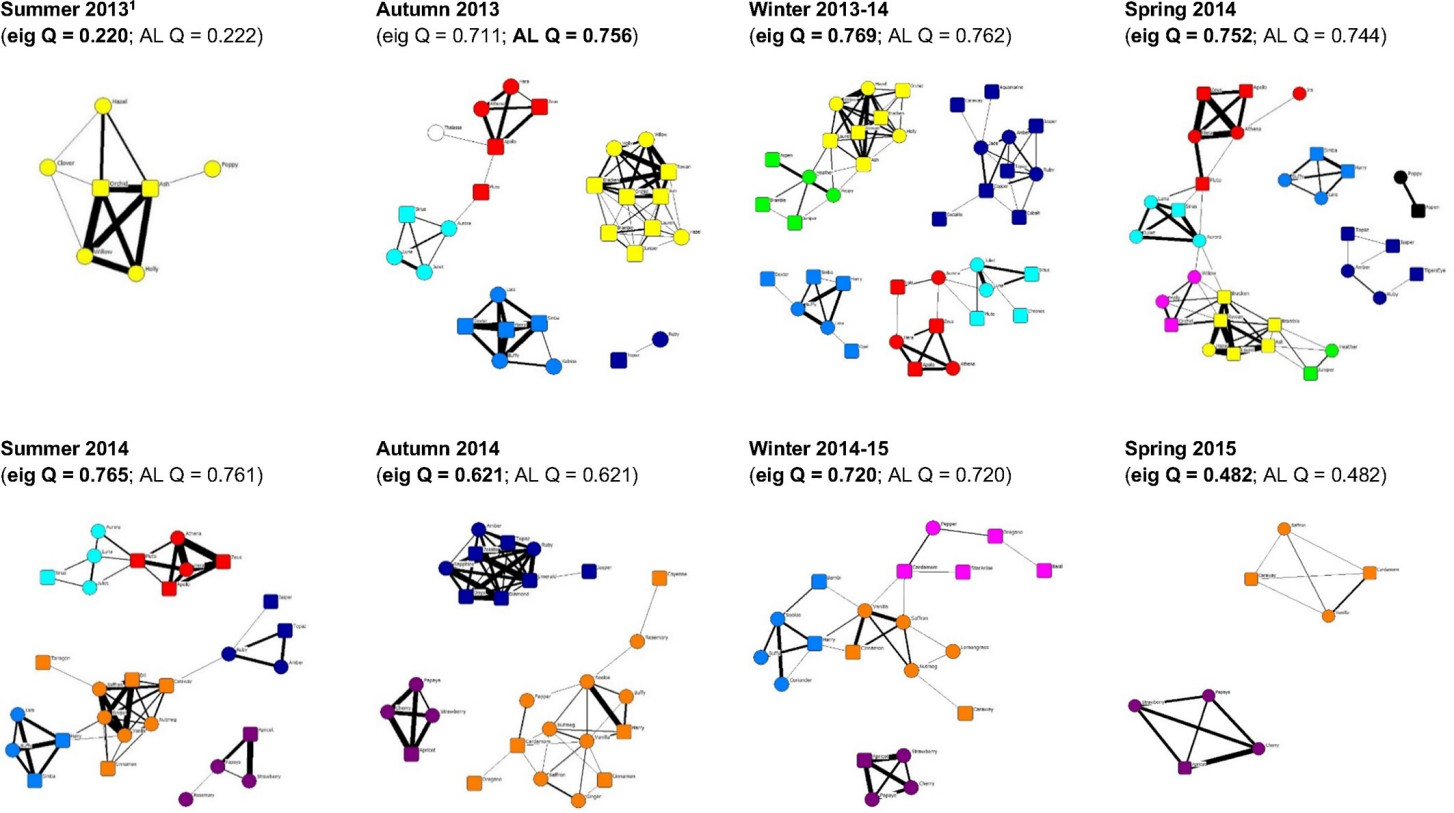
Consistency of communities in each season and year. Edge thickness is proportional to the simple ratio index. Node shapes represent males (■) and females (●). Eig Q = maximum modularity from eigenvector community detection, AL Q = maximum modularity from average-linkage hierarchical clustering. The communities presented are from the method in bold, based on the highest modularity or the eigenvector method when modularity was the same for both methods. ^1^ Q < 0.3 indicated the network could not be divided into communities so the full network is presented. The arrow indicates a male that switched communities between seasons.

Communities with numbers roughly matched the communities delineated from all seasons and years pooled (Table 2); season-year communities with no C-number did not match any community. Colours refer to those used in the network diagrams (Fig 1). M = male, F = female, Dom = dominant, Sub = subordinate, Ust = unknown social status.

Seventy foxes were interconnected in communities during at least one season across the two-year study (mean = 2.61, SD = 1.44, range = 1–6). Of these, 44 were observed in more than one season and 40 (91%) assigned to the same community in at least two seasons, though not always consecutively; 30 (68%) were assigned to the same community in every season they were observed (Fig 1). One subordinate male, marked with an arrow in Fig 1, temporarily switched communities in winter and community 1 was gradually subdivided into three separate communities.

**Fig 2:**
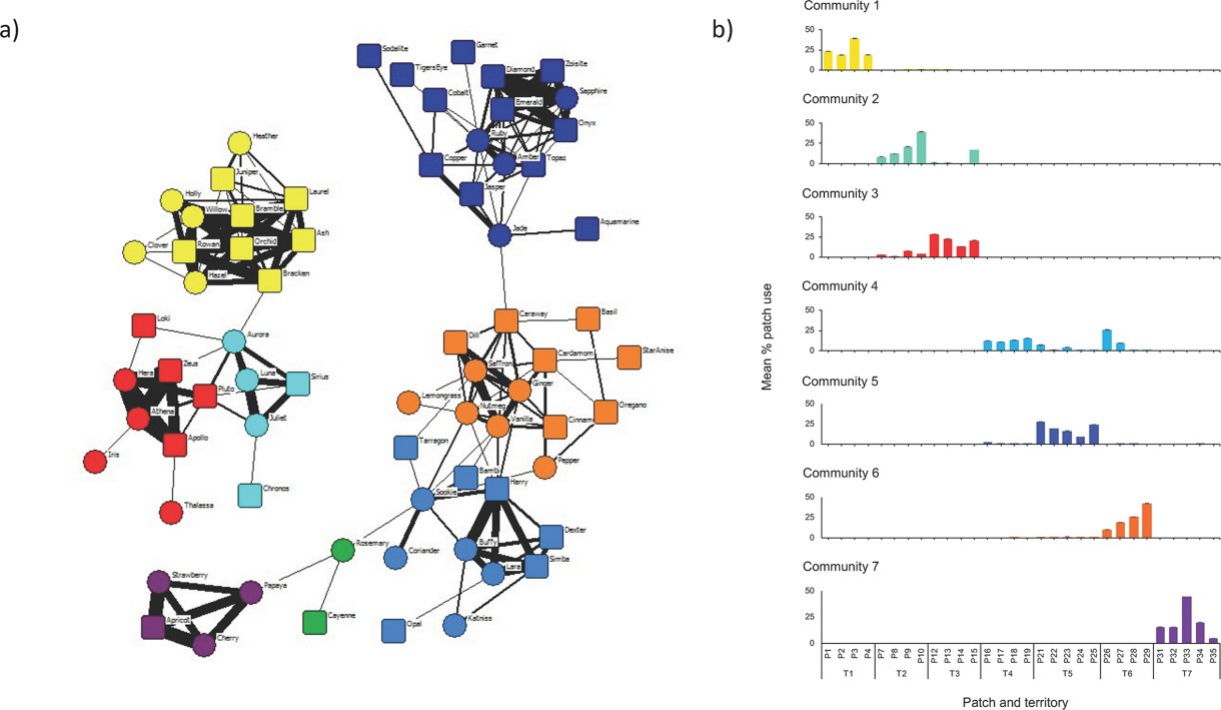
**(a) Communities 1–8 in the fox social network, excluding isolates.** Node shapes represent males (■) and females (●). Line thickness is proportional to the simple ratio index (SRI). (b) Spatial profiles of communities 1–7. Colours correspond to communities in (a) and error bars indicate standard deviation.

**Fig 3:**
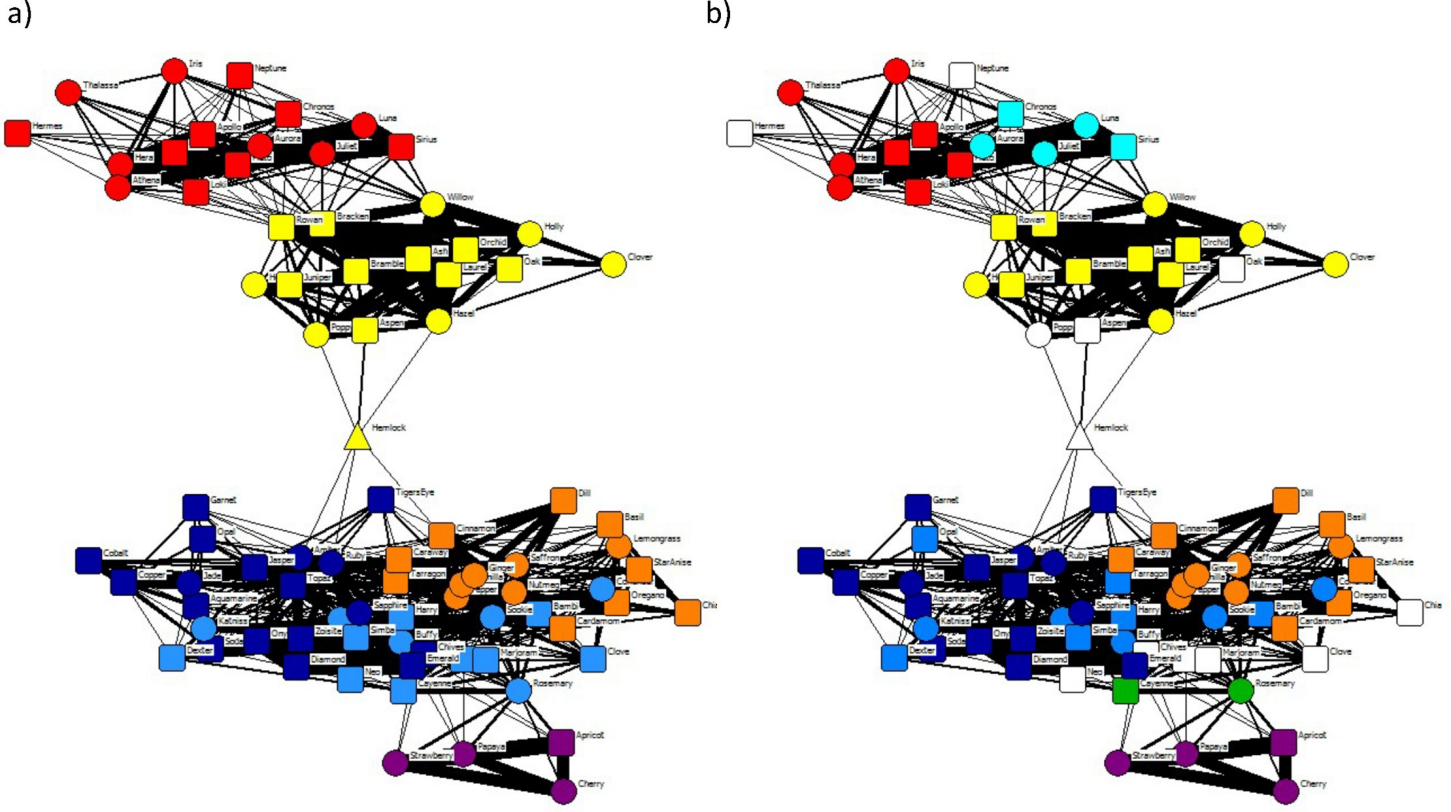
**Space use network with (a) colours representing communities based on daily space use overlap and (b) colours representing communities based on spatiotemporal associations, for comparison.** Line thickness is proportional to the simple ratio index (SRI) based on the number of days when dyads visited the same patches. Node shapes represent males (■), females (●) and unknown sex (▲).

True- and self-associations involving foxes in each community were recorded in multiple territories (Table 2), but most were observed in a single territory, as were the majority of patch visits by members of each community (Fig 2). Relationships were similar when defined by spatiotemporal associations and by same-day patch use (Dietz *R*-test: matrix rank correlation = 0.542, *p* < 0.001). However, eigenvector community detection split the population into fewer, larger communities when based on same-day patch use (Q = 0.715) compared to true spatiotemporal associations, and the resulting communities in this patch-use network included some individuals that were isolated in the social network (Fig 3).

**Fig 4:**
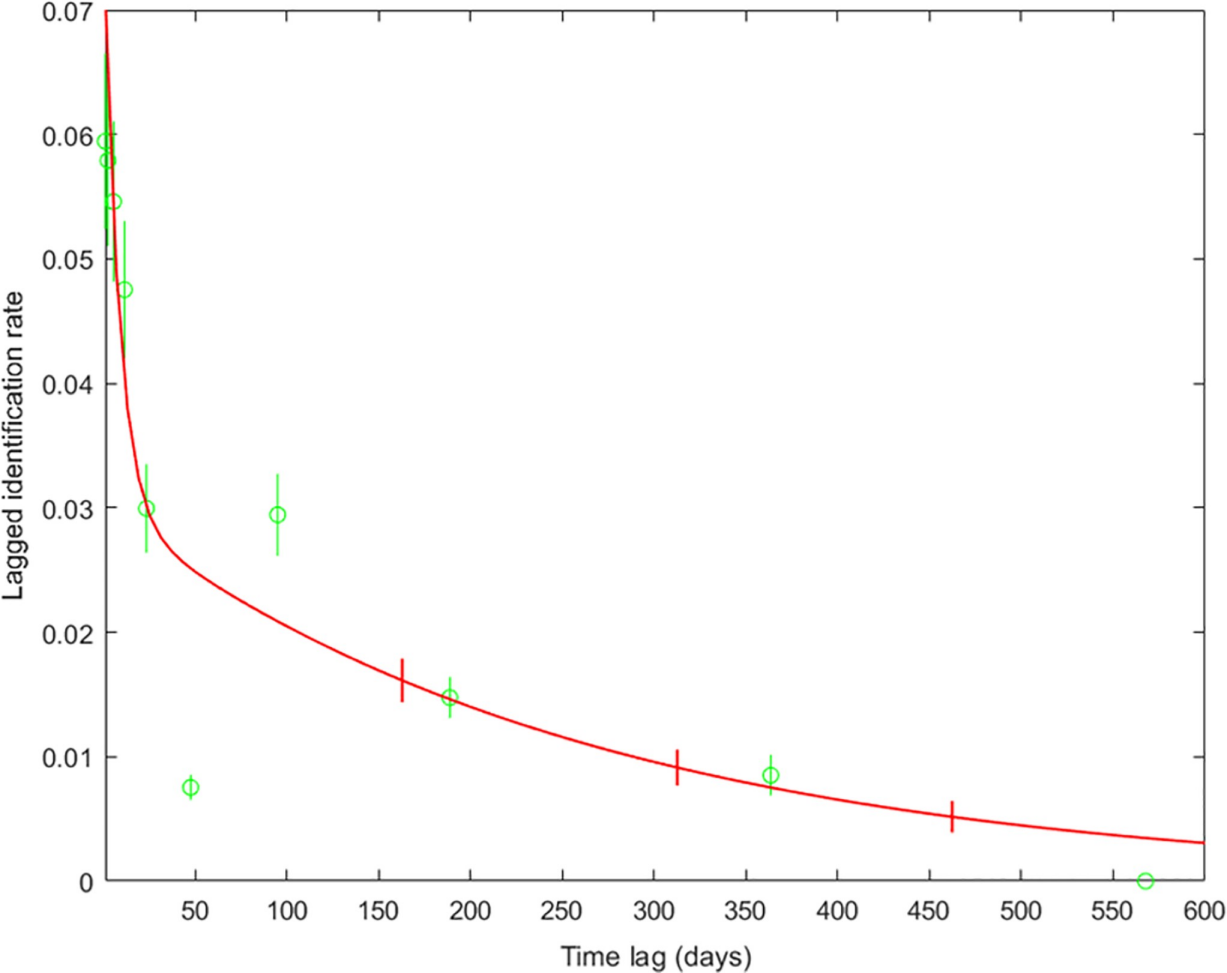
**The probability that a fox identified on a given day would be the same as a randomly chosen individual at a later time (lagged identification rate, LIR) across the whole study (green circles) and the best-fitting model (red line).** Error bars show bootstrap standard errors calculated over 100 replicates.

**Fig 5:**
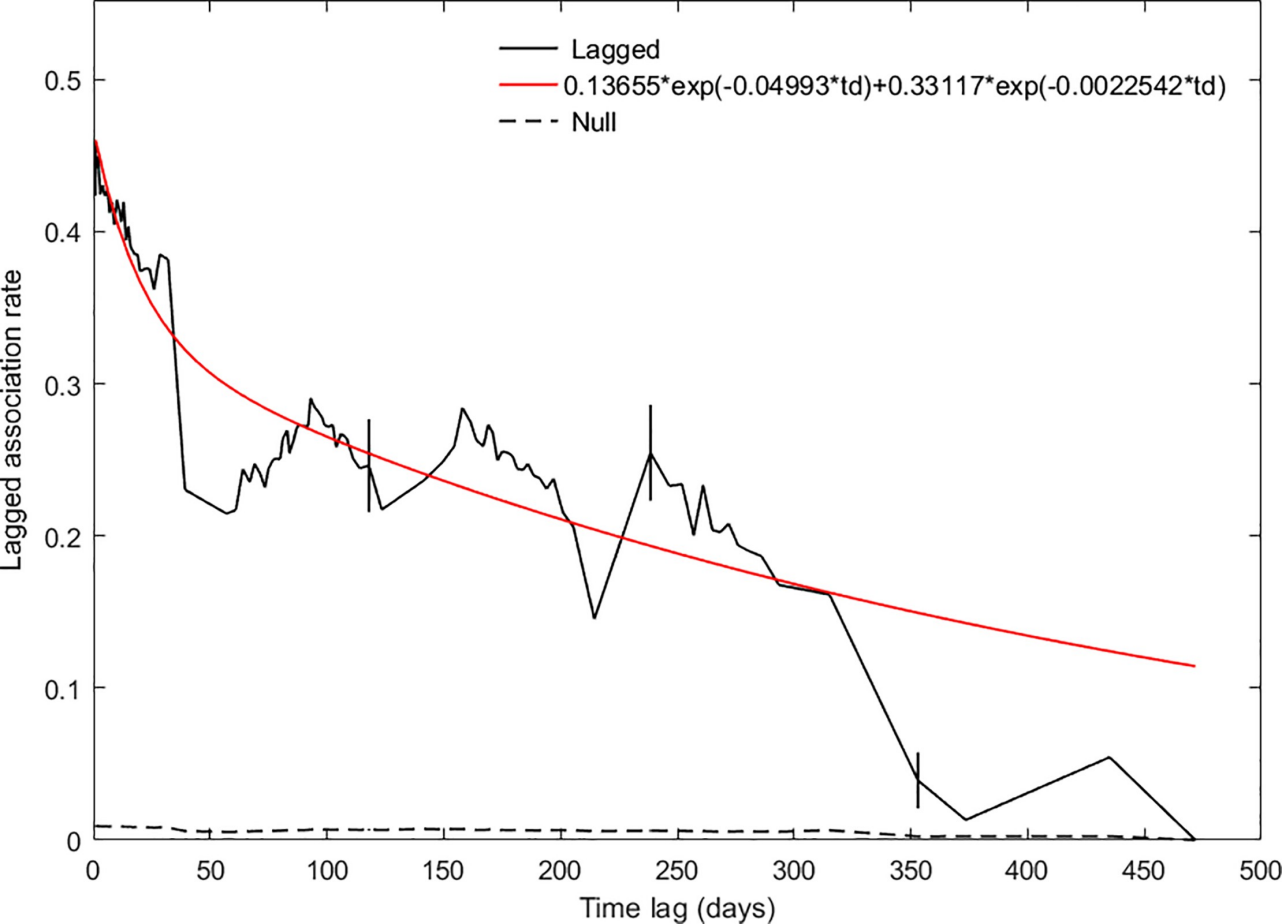
The probability that pairs of foxes that associated on a given day would re-associate at a later time (lagged association rate, LAR), the best-fitting exponential model and the expected association rate if associations were random (null association rate, NAR) for the whole dataset. Vertical lines show jack-knife standard errors.

The LIR plot (Fig 4) showed individuals identified in the study area over a 600-day period, the approximate length of the two-year study from July 2013 to May 2015. The best-fitting model included emigration, reimmigration and mortality (Table 4), when 13 of the 174 foxes in the analysis were identified on average for 14 days before ‘leaving’ the study area (i.e. not being identified) for 22 days, and then returning to the study area, with an estimated mortality rate of 0.0038/day (1 death every 263 days). As territories were not surveyed continuously, parameter estimates for movement in and out of the study area should be interpreted with caution. The shape of the steep decline in LIR (Fig 4) is more informative, as it matches the shape of the LAR (Fig 5).

**Table 4:** Best-fitting model parameter estimates and fitted model formula for the lagged identification rate, which included emigration, reimmigration and mortality.

Parameters	Estimate ± SE	95% CIs
N (a1)	13.0665 ± 1.667	10.768–16.777
Mean time in study area (a2)	13.9501 ± 1.186	13.102–16.577
Mean time out of study area (a3)	21.7136 ± 2.316	16.468–24.743
Mortality rate (a4)	0.0037999 ± 0.0006	0.002–0.005
Model formula: LIR = a3 × exp(−a1 × lag) + a4 × exp(−a2 × lag) Fitted function using estimated parameters: $LIR=\frac{exp(-0.0037999\times lag)}{13.0665}\times \frac{1/21.7136+1/13.9501\times \exp\left(-(1/21.7136+1/13.9501)\times lag\right)}{1/21.7136+1/13.9501}$		

Standard errors were estimated from 100 bootstrap replicates.

### Temporal stability of relationships

The LAR for the whole study period showed that foxes had periods of strong association lasting approximately 40 days, corresponding to the length of each survey (Fig 5). LARs declined between surveys, after which they increased again, almost to the previous level. This cyclic pattern is an artefact of the sampling regime and, when disregarding this pattern, the LAR declined at a rate comparable to the LIR (Fig 4). The LAR remained above the NAR for all time lags, indicating that foxes had preferred companionships at all time scales. The best-fitting model included permanent acquaintances (33% of relationships) projected to last 1 year 3 months, casual acquaintances (14%) that lasted for around 20 days, and rapid disassociations (53%) lasting less than a day (Table C in S2 Appendix).

Most LARs in communities and territories stayed above the NAR throughout the study, confirming the presence of stable and preferred companionships at all time scales (Figs D and E in S1 Appendix). LARs followed a similar downward trajectory in all communities and territories with the exceptions of communities/territories 3 and 7: they showed no apparent decline in association rate, suggesting greater long-term stability in these social groups. They also had the highest probability of re-association after a year (both probability > 0.5, Table C in S2 Appendix). LARs within territories were similar to those within their corresponding communities (Fig C in S1 Appendix), with the exception of community/territory 4. Relationships in this social unit lasted longer within the territory than the wider community, probably because associations between some community members were recorded in territory 6 (following the death of its dominant male) after territory 4 was surveyed, giving an erroneous impression that relationships with the other members of the community had broken down.

The best-fitting model for LARs in most communities and territories included a combination of rapid disassociations lasting less than a day, casual acquaintances lasting days or weeks and permanent preferred companions (Table C in S2 Appendix), although there were considerable differences in temporal association patterns between groups: 55% of associations were preferred companions in community/territory 3 but only 5% in community/territory 6. LARs in communities/territories 4 and 7 were better explained by rapid disassociation coupled with short- and long-term casual acquaintances. Parameter estimates were very similar for community and territory 7, with approximately 27% of associations lasting 26 days and 33% projected to last 500 days, whereas relationships appeared more stable in territory 4 than community 4 (Figs C and D in S1 Appendix).

**Fig 6:**
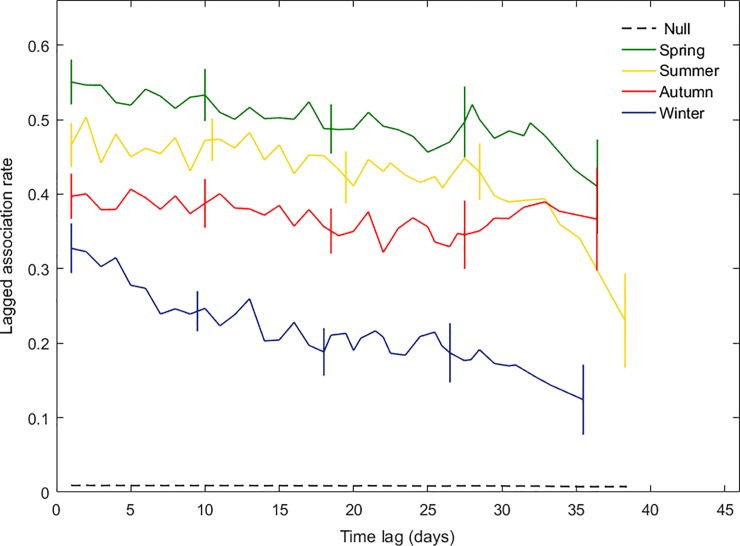
The probability that pairs of foxes that associated on a given day would re-associate at a later time (lagged association rate, LAR) within seasons (maximum lag = 40 days) and the expected association rate if associations were random (null association rate, NAR) calculated across all data. Vertical lines show jack-knife standard errors.

While within-season LARs declined slowly over the 40-day period, they did not cross the NAR, indicating that relationships were relatively stable within seasons (Fig 6). LARs were highest in spring and summer, and lowest in winter. The probability of re-association after 1 day (Table C in S2 Appendix) was highest in spring (0.49) and lowest in winter (0.34). The most parsimonious model for LARs in spring and summer included approximately equal proportions of rapid disassociations and casual acquaintances predicted to last 140–190 days (Table C in S2 Appendix). However, since territories were only monitored for 40 days per season, the model estimates of relationship duration assumed that foxes continued to associate in the same manner. In winter, relationships were best described as rapid disassociations (64%) and both short- (8%) and long-term (28%) casual acquaintances that lasted 3.8 days and 2 months, respectively. In autumn there were two top models with a similar goodness of fit. Both estimated that approximately 60% of associations lasted less than a day: one classed all remaining associations as casual acquaintances lasting 245 days, and the other split these into 34% preferred companions and 7% casual acquaintances lasting 24 days.

Patterns of association within territories were similar between seasons close in time (Table D in S2 Appendix). Combined *p*-values for all territories showed that association patterns were consistent over a maximum of three consecutive seasons (Table 5). The extent of similarity between association matrices in different seasons varied between territories e.g. they were more consistent in territories 1 and 3 than territory 4, where association patterns changed every season. No territory had significant similarities between all seasons.

**Table 5:** The consistency of association matrices in each territory in different seasons.

Territory	Time between seasons	χ^2^	df	*p*
T1	1	31.56	4	**<0.001**
	2	15.15	4	**0.004**
	3		1	0.056*
T2	1	8.76	4	0.067*
	2	8.91	4	0.064*
	3		1	0.287
T3	1	17.13	4	**0.002**
	2	11.00	4	**0.027**
	3		1	0.220
T4	1	8.17	4	0.086*
	2	6.07	4	0.194
	3		1	0.211
T5	1	15.60	4	**0.004**
	2	5.17	4	0.270
	3		1	0.127
T6	1	17.24	4	**0.002**
	2	10.16	4	**0.038**
	3		1	0.502
T7	1	4.73	4	0.316
	2	8.65	4	0.071*
	3		1	0.500
All	1	103.19	28	**<0.001**
	2	65.12	28	**<0.001**
	3	21.30	14	0.094*

Dietz-*R* Mantel test *p-*values were combined by Fisher's Omnibus Test. The time between seasons indicates how close in time the compared surveys were: 1 = consecutive, 2 = gap of one season and 3 = gap of 2 seasons. Significant values (*p* < 0.05) and those close to significance (0.1 > *p* > 0.05, marked with *) are shown in bold.

## Discussion

Although camera traps are rarely used to study animal social systems [86,87], we have demonstrated that they can collect unbiased data from multiple social units for continuous time periods: while the number of sites that can be monitored is limited, camera traps record associations between all members of a population, which is rarely possible with proximity loggers or passive integrated transponder (PIT) tags.

Despite the widespread perception that red foxes are in some way primitively social [50], we have shown that foxes have a highly differentiated society: individuals have short- and long-term relationships and a community structure probably explained by territoriality. Although facultatively social species generally meet few conspecifics when foraging e.g. [88], the foxes on our study area encountered a conspecific on 13% of patch visits, which was comparable to encounters when travelling between foraging patches on the same study site [52], although these data are from a period when social group sizes were smaller. However, we caution that our data were based on visits to high-quality foraging patches: it is unclear how often different members of each social group use less productive foraging patches, and whether there is a social bias so that less productive patches are used more often by lower-ranking group members [61]. Furthermore, while foxes forage alone, they often share rendezvous sites during the day when cub rearing [89] and at other times of the year. Thus adult members of the social group may spend much of each day in close proximity, although it is unclear whether status and/or other factors affect the frequency with which different members of the social group use rendezvous sites. However, when testing within territories, foxes had preferred and avoided companions both within and between days in all seasons other than summer, when the only preferences were between-day. Intragroup encounters were more common than intergroup encounters [52], and territory intrusions by non-residents were lowest in summer [50], which may explain the lack of within-day avoided companionships.

### Foxes associated in communities within a territory boundary

Our fox population was divided into 7 high-confidence communities containing 4–16 individuals that associated with each other more often than with individuals in other communities, suggesting that they associated with up to 15 conspecifics more often than occasionally. Community structure consisted of a dominant pair with several subordinates and an approximately equal sex ratio [89]. However, communities spanned the whole study period and several members were not present for the entire duration of the study. Each fox was only assigned to one community, and communities contained far fewer individuals than territories, since for many carnivores some individuals (particularly males) visit food patches in more than one territory [57,61,90–93]. Territories have long been assumed to match social groups in Bristol [64] and, as expected, community structure largely matched territorial space use. The majority of patch visits by members of the same community were recorded at patches in the same territory, and networks of spatiotemporal associations were statistically similar to networks of same-day patch use. Furthermore, the longevity of relationships was comparable between matched territories and communities, confirming that foxes mainly associated within their territory boundary and that social groups defined by shared territory use [49,62,94] are accurate.

Community structure has been linked to space use in other studies that used the ‘gambit of the group’ approach to infer social associations [19,95–98]. However, it is possible that the fox communities we detected arose from shared space use rather than preferential social associations since these factors are difficult to differentiate [21,99]. We were unable to customise null models to test the significance of association patterns while controlling for sighting location [100] or the extent of space use overlap [20]. Nevertheless, visual inspection showed that fox communities based solely on same-day shared space use (foxes seen in the same patch on the same day, but not at the same time) were larger than communities based on spatiotemporal associations, contained individuals that were isolated in the social network, and grouped many dyads that had never visited the same patch.

Of the 70 foxes seen in more than one season, 44 (68%) were assigned to the same community in all seasons, indicating that community structure was associated with space use for most foxes [19,97]. Community 1 was gradually split into three separate communities; most of the other foxes that changed community were subordinates and did so in autumn or winter, when increased extraterritorial movements [57] lead to higher inter-group encounter rates [52]. This may make social groups appear less distinct: communities are far easier to define when connections are rare between, and common within, groups [101]. It could also be that fox communities overlap [102], but this seems unlikely in a territorial species.

### Temporal stability of red fox relationships

The probability that an individual was re-identified after a given time lag (LIR) declined steeply in the 40 days following their initial sighting, and continued to drop, albeit at a slower rate, throughout the study period. Infrequent non-resident visitors, and individuals only identified in one survey, probably accounted for the initial steep drop. While the LIR suggested that foxes followed a cyclical pattern of emigration and reimmigration, this was largely down to our sampling protocol. LARs showed a similar cyclical pattern, with high rates of re-association during surveys and low rates between surveys: their rise at the beginning of each subsequent survey indicated that foxes had preferred companions that were consistent between consecutive surveys. However, the LAR declined steadily over time and, as this matched the LIR, this was probably due to dispersal or mortality rather than a breakdown of social relationships [81]. This was further supported by pairwise comparisons between association matrices in different seasons, which confirmed that relationships were stable over seasons, suggesting that, contrary to [51], changes in patterns of association did not occur at particular times of the year.

We identified three main types of relationships: 33% were long term (persisted throughout all four seasons in a territory), 14% were short term (lasting for 20 days based on the combined dataset, but 12–66 days depending on the community/territory), and 53% were rapid disassociations lasting less than a day. Thus foxes have long-term relationships with other territory residents, associate less often with more peripheral residents or foxes from neighbouring territories, and associate just once, if at all, with individuals such as dispersers or foxes seeking mating opportunities that visit their territory for short periods. Most dyads only associated once during the study and were probably between foxes in different social groups. Although inter-group associations were less common than intra-group associations, and did not persist for as long, they may be more common than previously thought [52]. Around 73% of relationships in community/territory 5 were rapid disassociations because many individuals were connected to the network by a single or weak association that did not represent a strong social bond, highlighting the importance of considering association strength when defining fox social groups based on spatiotemporal associations [50].

The stability and distribution of types of relationships differed between unmatched territories and communities. Between-community variation is not uncommon [19,103,104] and probably indicates the influence of multiple interacting variables on social behaviour, although it could be related to network size: smaller communities are more stable temporally [105]. Relationships were most stable in two of the smallest communities, which showed no decline in LAR throughout the year they were studied, and had the highest proportion of long-term companionships (55% in community/territory 3, 33% in community/territory 7). Community/territory 6 had the lowest proportion of long-term companionships (< 5% relationships), further demonstrating the impact of the death of the dominant male on social stability. In the first survey in territory 6 (summer 2014, days 1–40), when the dominant male was alive, LARs followed a similar trajectory to the other communities/territories, but in the second survey (autumn 2014, days 80–120) which followed his death, LARs dropped below all the other territories and fell to random after 275 days (spring 2015), once a new dominant male had become established and one of the long-term resident subordinate females, the only remaining long-term companion of the original dominant female, had died. At that point no other fox had been resident in territory 6 since the first survey. Despite their social flexibility, the loss of a group member had a larger and longer-lived impact on fox social structure than expected. Opportunities to study the effects of individual removal on social structure are rare in wild populations and so are generally only examined using simulations [10,21,44,106–109].

LARs were relatively constant within, but differed between, seasons. They were highest in spring and summer and lowest in winter, when true associations were least common, suggesting a seasonal variation in social connectivity, or cohesion. In spring and summer there were equal proportions of rapid disassociations and long-term relationships, whereas long-term relationships were less common in autumn (34–40%) and most relationships lasted less than a day. Long-term relationships were least common in winter (28%), when most relationships lasted less than a day. Foxes maintained preferred companionships with other territory residents at the onset of dispersal, but association rates with dispersing residents declined and the increase in territory intrusion by non-resident dispersers and foxes seeking mating opportunities led to a greater number of short-lived inter-group contacts: mammalian sociality typically declines at the onset of dispersal [25,30].

In future, examining the effects of individual attributes on LARs could help explain the observed patterns of temporal stability. Sex and age differences have been reported in the longevity of relationships in other species [11,110–113], and juveniles may also be important for maintaining social connectivity in red foxes: data on relatedness could also help explain preferred associations [87].

## Conclusions

This is one of the first studies to apply social network analysis to red foxes and to camera trap data. Red foxes have a highly differentiated society with a community structure explained by territorial space use. Relationships were mostly stable, but for a limited period of time due to birth, death and dispersal, leading to alterations in social structure. Foxes maintained long-term companionships with some conspecifics in their home territory that lasted several seasons until dispersal or mortality. However, over half of all relationships lasted less than a day, particularly during the dispersal and mating seasons, and these were probably between foxes in different social groups. Furthering our understanding of inter-group relationships is a major challenge in behavioural ecology [114] and, from an applied perspective, are fundamental to improving models for population management and disease spread in red foxes e.g. [115]. Hitherto, management models have focussed on population processes e.g. [116]. While extremely valuable in helping inform management decisions, there is currently a dearth of data on social interactions in red foxes, and the social and population effects of management interventions. The data presented here, particularly on the high levels of inter-group social links, will help improve the management of this globally important invasive species [117]. However, we caution that, of necessity, we collected data from the key foraging patches in each territory. Social connections are likely to vary with context [118] and our data relate to a specific activity.

## Supporting information

S1 Appendix **Fig A. Distributions of sighting frequencies (days observed out of 40) for foxes > 5 months old.** Distributions are plotted separately for each season and for all data pooled.
**Fig B. The proportion of dyads with simple ratio association indices (SRI) of increasing strength for individuals seen on ≥ 5 days in each season and territory.** The proportion of individuals is on the y-axis and SRI, plotted between 0–1, is on the x-axis.
**Fig C. Proportions of individuals seen on ≥ 5 days in each territory and season with mean (blue) and maximum (yellow) simple ratio association indices (SRI) of increasing strength.** Proportion of individuals is on the y-axis and SRI, plotted between 0–1, is on the x-axis.
**Fig D. The probability that pairs of foxes that associated on a given day would re-associate at a later time (lagged association rate, LAR) within communities 1–7, and the expected association rate if associations were random (null association rate, NAR) calculated across all data.** Vertical lines show jack-knife standard errors.
**Fig E. The probability that pairs of foxes that associated on a given day would re-associate at a later time (lagged association rate, LAR) within each territory, and the expected association rate if associations were random (null association rate, NAR) calculated across all data.** Vertical lines show jack-knife standard errors.
(DOCX)

S2 Appendix **Table A. Summary of survey effort and association data used to construct social networks.** T = territory; days = sampling periods; patches = camera sites; observations = observations of filtered individuals; true associations = dyadic associations rather than self-associations; *S* = maximum likelihood estimate of social differentiation; *r* = correlation between true and estimated association indices; SE = standard error. Bold values indicate *S* ≥ 0.2 or *r* ≥ 0.4. *—some patches were not used for all four seasons.
**Table B. Results of the Manly/Bejder tests for preferred and avoided companions for the combined dataset and separately for each territory and season network.** Dashes indicate degenerate networks that were too sparse to permute. * indicates the *p*-value was close to significance (0.1 > *p* > 0.05).
**Table C. Parameter estimates and interpretation of the best-fitting exponential decay models fitted to lagged association rates (LARs) for the whole dataset and separate seasons, communities and territories.** Standard errors were estimated by jack-knifing over one day. The models with the lowest QAIC each contained proportions of rapid disassociations (RD), preferred companions (PC) and casual acquaintances (CA) of one or two types, defined in the formulae as a1-a4. Lag = time lag in days. The top two models are presented for autumn as ΔQAIC < 2. AR indicates the probability of re-association after given time lags in days.
**Table D. Mantel test correlations (*R*) between seasonal association matrices with the mean *p*-value and standard deviation (SD) calculated from three runs each with 10,000 permutations.** Season pair denotes the two surveys compared, listed in the order of data collection: spring (SP), summer (SU), autumn (AU) and winter (WI). Time between seasons indicates how close in time the compared surveys were: 1 = consecutive, 2 = gap of one season and 3 = gap of 2 seasons. Significant *p*-values (*p* < 0.05) and those close to significance (0.1 > *p* > 0.05, marked with *) are shown in bold.
(DOCX)

## References

[1] Ekernas LS, Cords M. Social and environmental factors influencing natal dispersal in blue monkeys, *Cercopithecus mitis stuhlmanni*. Anim Behav. 2007;73: 1009–1020. 10.1016/j.anbehav.2006.11.007.

[2] Blumstein DT, Wey TW, Tang K. A test of the social cohesion hypothesis: interactive female marmots remain at home. Proc R Soc B. 2009;276: 3007–3012. 10.1098/rspb.2009.0703 19493901 PMC2817218

[3] Cameron EZ, Setsaas TH, Linklater WL. Social bonds between unrelated females increase reproductive success in feral horses. Proc Nat Acad Sci. 2009;106: 13850–13853. 10.1073/pnas.0900639106 19667179 PMC2728983

[4] Holt-Lunstad J, Smith TB, Layton JB. Social relationships and mortality risk: a meta-analytic review. PLoS Med. 2010;7(7): e1000316 10.1371/journal.pmed.1000316 20668659 PMC2910600

[5] Schülke O, Bhagavatula J, Vigilant L, Ostner J. Social bonds enhance reproductive success in male macaques. Current Biol. 2010;20: 2207–2210. 10.1016/j.cub.2010.10.058. 21093261

[6] Wey TW, Blumstein DT. Social attributes and associated performance measures in marmots: bigger male bullies and weakly affiliating females have higher annual reproductive success. Behav Ecol Sociobiol. 2012;66: 1075–1085. 10.1007/s00265-012-1358-8.

[7] Wiszniewski J, Corrigan S, Beheregaray LB, Möller LM. Male reproductive success increases with alliance size in Indo-Pacific bottlenose dolphins (*Tursiops aduncus*). J Anim Ecol. 2012;81: 423–431. 10.1111/j.1365-2656.2011.01910.x 21981240

[8] Hoogland JL. Prairie dogs disperse when all close kin have disappeared. Science. 2013;339: 1205–1207. 10.1126/science.1231689 23471407

[9] Borg BL, Brainerd SM, Meier TJ, Prugh LR. Impacts of breeder loss on social structure, reproduction and population growth in a social canid. J Anim Ecol. 2015;84: 177–187. 10.1111/1365-2656.12256 25041127

[10] Kurvers RHJM, Krause J, Croft DP, Wilson ADM, Wolf M. The evolutionary and ecological consequences of animal social networks: emerging issues. Trends Ecol Evol. 2014;29: 326–335. 10.1016/j.tree.2014.04.002 24792356

[11] Podgórski T, Lusseau D, Scandura M, Sönnichsen L, Jędrzejewska B. Long-lasting, kin-directed female interactions in a spatially structured wild boar social network. PLoS One. 2014;9(6): e99875 10.1371/journal.pone.0099875 24919178 PMC4053407

[12] Vander Wal E, Festa-Bianchet M, Réale D, Coltman DW, Pelletier F. Sex-based differences in the adaptive value of social behavior contrasted against morphology and environment. Ecology. 2015;96: 631–641. 10.1890/14-1320.1. 26236860

[13] Farine DR, Whitehead H. Constructing, conducting and interpreting animal social network analysis. J Anim Ecol. 2015;84: 1144–1163. 10.1111/1365-2656.12418 26172345 PMC4973823

[14] Whitehead H. Analyzing animal societies: quantitative methods for vertebrate social analysis. Chicago: University of Chicago Press; 2008.

[15] Pepper JW, Mitani JC, Watts DP. General gregariousness and specific social preferences among wild chimpanzees. Int J Primat. 1999;20: 613–632. 10.1023/A:1020760616641.

[16] Girvan M, Newman MEJ. Community structure in social and biological networks. Proc Nat Acad Sci. 2002;99: 7821–7826. 10.1073/pnas.122653799 12060727 PMC122977

[17] Dunbar RIM. The social brain hypothesis. Evol Anthropol. 1998;6: 178–190. 10.1002/(SICI)1520-6505(1998)6:5<178::AID-EVAN5>3.0.CO;2-8.

[18] Wolf JBW, Mawdsley D, Trillmich F, James R. Social structure in a colonial mammal: unravelling hidden structural layers and their foundations by network analysis. Anim Behav. 2007;74: 1293–1302. 10.1016/j.anbehav.2007.02.024.

[19] Wiszniewski J, Allen SJ, Möller LM. Social cohesion in a hierarchically structured embayment population of Indo-Pacific bottlenose dolphins. Anim Behav. 2009;77: 1449–1457. 10.1016/j.anbehav.2009.02.025.

[20] Best EC, Dwyer RG, Seddon JM, Goldizen AW. Associations are more strongly correlated with space use than kinship in female eastern grey kangaroos. Anim Behav. 2014;89: 1–10. 10.1016/j.anbehav.2013.12.011.

[21] Pinter-Wollman N, Hobson EA, Smith JE, Edelman AJ, Shizuka D, de Silva S, et al The dynamics of animal social networks: analytical, conceptual, and theoretical advances. Behav Ecol. 2014;25: 242–255. 10.1093/beheco/art047.

[22] Verdolin JL, Traud AL, Dunn RR. Key players and hierarchical organization of prairie dog social networks. Ecol Complex. 2014;19: 140–147. 10.1016/j.ecocom.2014.06.003.

[23] Whitehead H, Dufault S. Techniques for analyzing vertebrate social structure using identified individuals: review and recommendations. Adv Study Behav. 1999;28: 33–74.

[24] Hamede RK, Bashford J, McCallum H, Jones M. Contact networks in a wild Tasmanian devil (*Sarcophilus harrisii*) population: using social network analysis to reveal seasonal variability in social behaviour and its implications for transmission of devil facial tumour disease. Ecol Lett. 2009;12: 1147–1157. 10.1111/j.1461-0248.2009.01370.x 19694783

[25] Patriquin KJ, Leonard ML, Broders HG, Garroway CJ. Do social networks of female northern long-eared bats vary with reproductive period and age? Behav Ecol Sociobiol. 2010;64: 899–913. 10.1007/s00265-010-0905-4.

[26] Brent LJN, MacLarnon A, Platt ML, Semple S. Seasonal changes in the structure of rhesus macaque social networks. Behav Ecol Sociobiol. 2013;67: 349–359. 10.1007/s00265-012-1455-8 23565026 PMC3613995

[27] Wey TW, Burger JR, Ebensperger LA, Hayes LD. Reproductive correlates of social network variation in plurally breeding degus (*Octodon degus*). Anim Behav. 2013;85: 1407–1414. 10.1016/j.anbehav.2013.03.035 24511149 PMC3914217

[28] Reynolds JJH, Hirsch BT, Gehrt SD, Craft ME. Raccoon contact networks predict seasonal susceptibility to rabies outbreaks and limitations of vaccination. J Anim Ecol. 2015;84: 1720–1731. 10.1111/1365-2656.12422 26172427

[29] Smith JE, Kolowski JM, Graham KE, Dawes SE, Holekamp KE. Social and ecological determinants of fission-fusion dynamics in the spotted hyaena. Anim Behav. 2008;76: 619–636. 10.1016/j.anbehav.2008.05.001.

[30] Henzi SP, Lusseau D, Weingrill T, van Schaik CP, Barrett L. Cyclicity in the structure of female baboon social networks. Behavl Ecol Sociobiol. 2009;63: 1015–1021. 10.1007/s00265-009-0720-y.

[31] Foster EA, Franks DW, Morrell LJ, Balcomb KC, Parsons KM, van Ginneken A, et al Social network correlates of food availability in an endangered population of killer whales, *Orcinus orca*. Anim Behav. 2012;83: 731–736. 10.1016/j.anbehav.2011.12.021.

[32] Duboscq J, Romano V, Sueur C, MacIntosh AJJ. Network centrality and seasonality interact to predict lice load in a social primate. Sci Rep. 2016;6: 22095 10.1038/srep22095 26915589 PMC4768153

[33] de Silva S, Ranjeewa ADG, Kryazhimskiy S. The dynamics of social networks among female Asian elephants. BMC Ecol. 2011;11: 17 10.1186/1472-6785-11-17 21794147 PMC3199741

[34] Webster MM, Atton N, Hoppitt WJE, Laland KN. Environmental complexity influences association network structure and network-based diffusion of foraging information in fish shoals. Am Nat. 2013;181: 235–244. 10.1086/668825 23348777

[35] Webber QMR, Brigham RM, Park AD, Gillam EH, O’Shea TJ, Willis CKR. Social network characteristics and predicted pathogen transmission in summer colonies of female big brown bats (*Eptesicus fuscus*). Behav Ecol Sociobiol. 2016;70: 701–712. 10.1007/s00265-016-2093-3.

[36] Whitehead H. Investigating structure and temporal scale in social organizations using identified individuals. Behav Ecol. 1995;6: 199–208. 10.1093/beheco/6.2.199.

[37] Wallach AD, Johnson CN, Ritchie EG, O’Neill AJ. Predator control promotes invasive dominated ecological states. Ecol Lett. 2010;13: 1008–1018. 10.1111/j.1461-0248.2010.01492.x 20545732

[38] Shannon G, Slotow R, Durant SM, Sayialel KN, Poole J, Moss C, et al Effects of social disruption in elephants persist decades after culling. Front Zool. 2013;10: 62 10.1186/1742-9994-10-62 24152378 PMC3874604

[39] Spencer PBS, Hampton JO, Pacioni C, Kennedy MS, Saalfeld K, Rose K, et al Genetic relationships within social groups influence the application of the Judas technique: a case study with wild dromedary camels. J Wildl Manage. 2015;79: 102–111. 10.1002/jwmg.807.

[40] Doherty TS, Ritchie EG. Stop jumping the gun: a call for evidence-based invasive predator management. Conserv Lett. 2016;10: 15–22. 10.1111/conl.12251.

[41] Drewe JA. Who infects whom? Social networks and tuberculosis transmission in wild meerkats. Proc R Soc B. 2010;277: 633–642. 10.1098/rspb.2009.1775 19889705 PMC2842696

[42] Lusseau D, Newman MEJ. Identifying the role that animals play in their social networks. Proc R Soc B. 2004;271: S477–S481. 10.1098/rsbl.2004.0225 15801609 PMC1810112

[43] Flack JC, Girvan M, de Waal FBM, Krakauer DC. Policing stabilizes construction of social niches in primates. Nature, 2006;439: 426–429. 10.1038/nature04326 16437106

[44] Bret C, Sueur C, Ngoubangoye B, Verrier D, Deneubourg J-L, Petit O. Social structure of a semi-free ranging group of mandrills (*Mandrillus sphinx*): a social network analysis. PLoS One. 2013;8(12): e83015 10.1371/journal.pone.0083015 24340074 PMC3858359

[45] Schipper J, Chanson JS, Chiozza F, Cox NA, Hoffmann M, Katariya V, et al The status of the world’s land and marine mammals: diversity, threat, and knowledge. Science. 2008;322: 225–230. 10.1126/science.1165115 18845749

[46] Cavallini P. Variation in the social system of the red fox. Ethol Ecol Evol. 1996;8: 323–342. 10.1080/08927014.1996.9522906.

[47] Baker PJ, Harris S. Red foxes: the behavioural ecology of red foxes in urban Bristol In: Macdonald DW, Sillero-Zubiri C, editors. Biology and conservation of wild canids. Oxford: Oxford University Press; 2004 pp. 207–216.

[48] Baker PJ, Funk SM, Bruford MW, Harris S. Polygynandry in a red fox population: implications for the evolution of group living in canids? Behav Ecol. 2004;15: 766–778. 10.1093/beheco/arh077.

[49] Iossa G, Soulsbury CD, Baker PJ, Edwards KJ, Harris S. Behavioral changes associated with a population density decline in the facultatively social red fox. Behav Ecol. 2009;20: 385–395. 10.1093/beheco/arn149.

[50] Dorning J, Harris S. Quantifying group size in facultatively social species: what, if anything, is a fox social group? J Zool. 2019;308: 37–46.

[51] Poulle ML, Artois M, Roeder JJ. Dynamics of spatial relationships among members of a fox group (*Vulpes vulpes*: Mammalia: Carnivora). J Zool. 1994;233: 93–106. 10.1111/j.1469-7998.1994.tb05264.x.

[52] White PCL, Harris S. Encounters between red foxes (*Vulpes vulpes*): implications for territory maintenance, social cohesion and dispersal. J Anim Ecol. 1994;63: 315–327. 10.2307/5550

[53] Harris S, Smith GC. Demography of two urban fox (*Vulpes vulpes*) populations. J Appl Ecol. 1987;24: 75–86. 10.2307/2403788

[54] Harris S, Trewhella WJ. An analysis of some of the factors affecting dispersal in an urban fox (*Vulpes vulpes*) population. J Appl Ecol. 1988;25: 409–422. 10.2307/2403833

[55] Saunders G, White PCL, Harris S, Rayner JMV. Urban foxes (*Vulpes vulpes*): food acquisition, time and energy budgeting of a generalized predator. Symp Zool Soc Lond. 1993;65: 215–234.

[56] Baker PJ, Newman T, Harris S. Bristol’s foxes—40 years of change. British Wildl. 2001;12: 411–417.

[57] Soulsbury CD, Iossa G, Baker PJ, White PCL, Harris S. Behavioral and spatial analysis of extraterritorial movements in red foxes (*Vulpes vulpes*). J Mammal. 2011;92: 190–199. 10.1644/09-MAMM-A-187.1

[58] Lloyd HG. The red fox. London: Batsford; 1980.

[59] White PCL, Saunders G, Harris S. Spatio-temporal patterns of home range use by foxes (*Vulpes vulpes*) in urban environments. J Anim Ecol. 1996;65: 121–125. 10.2307/5705

[60] Baker PJ, Funk SM, Harris S, White PCL. Flexible spatial organization of urban foxes, *Vulpes vulpes*, before and during an outbreak of sarcoptic mange. Anim Behav. 2000; 59: 127–146. 10.1006/anbe.1999.1285 10640375

[61] Dorning J, Harris S. Dominance, gender, and season influence food patch use in a group-living, solitary foraging canid. Behav Ecol. 2017;28: 1302–1313. 10.1093/beheco/arx092

[62] Harris S. An estimation of the number of foxes (*Vulpes vulpes*) in the city of Bristol, and some possible factors affecting their distribution. J Appl Ecol. 1981;18: 455–465. https://www.jstor.org/stable/2402406.

[63] Soulsbury CD, Iossa G, Baker PJ, Cole NC, Funk SM, Harris S. The impact of sarcoptic mange *Sarcoptes scabiei* on the British fox *Vulpes vulpes* population. Mammal Rev. 2007;37: 278–296. 10.1111/j.1365-2907.2007.00100.x.

[64] Whiteside H. The role of subordinate reproduction on the promotion of group living in the red fox (*Vulpes vulpes*). Ph.D. Thesis, The University of Bristol. 2012.

[65] Dorning J, Harris S. The challenges of recognising individuals of species with few distinguishing features: Identifying red foxes *Vulpes vulpes* from camera trap photos. PLoS One. 2019;14(5): e0216531 10.1371/journal.pone.0216531 31071143 PMC6508734

[66] Perkins SE, Cagnacci F, Stradiotto A, Arnoldi D, Hudson PJ. Comparison of social networks derived from ecological data: implications for inferring infectious disease dynamics. J Anim Ecol. 2009;78: 1015–1022. 10.1111/j.1365-2656.2009.01557.x 19486206

[67] Castles M, Heinsohn R, Marshall HH, Lee AEG, Cowlishaw G, Carter AJ. Social networks created with different techniques are not comparable. Anim Behav. 2014;96: 59–67. 10.1016/j.anbehav.2014.07.023.

[68] Whitehead H. SOCPROG programs: analysing animal social structures. Behav Ecol Sociobiol. 2009;63: 765–778. 10.1007/s00265-008-0697-y.

[69] Bejder L, Fletcher D, Bräger S. A method for testing association patterns of social animals. Anim Behav. 1998;56: 719–725. 10.1006/anbe.1998.0802 9784222

[70] Cairns SJ, Schwager SJ. A comparison of association indices. Anim Behav. 1987;35: 1454–1469. 10.1016/S0003-3472(87)80018-0.

[71] Ginsberg JR, Young TP. Measuring association between individuals or groups in behavioural studies. Anim Behav. 1992;44: 377–379.

[72] Whitehead H. Precision and power in the analysis of social structure using associations. Anim Behav. 2008;75: 1093–1099. 10.1016/j.anbehav.2007.08.022.

[73] Borgatti SP. NetDraw software for network visualization. Lexington, KY: Analytic Technologies; 2002.

[74] Manly BFJ. A note on the analysis of species co-occurrences. Ecology 1995;76: 1109–1115. 10.2307/1940919.

[75] Whitehead H. Testing association patterns of social animals. Anim Behav. 1999;57: 26–29. 10.1006/anbe.1999.1099 10373270

[76] Newman MEJ. Modularity and community structure in networks. Proc Nat Acad Sci. 2006;103: 8577–8582. 10.1073/pnas.0601602103 16723398 PMC1482622

[77] Lusseau D. Why are male social relationships complex in the Doubtful Sound bottlenose dolphin population? PLoS One. 2007;2(4): e348 10.1371/journal.pone.0000348 17406672 PMC1831491

[78] Newman MEJ. Analysis of weighted networks. Phys Rev E, 2004;70(5): 056131 10.1103/PhysRevE.70.056131. 15600716

[79] Dietz EJ. Permutation tests for association between two distance matrices. Syst Biol. 1983;32: 21–26. 10.1093/sysbio/32.1.21.

[80] Whitehead H. Analysis of animal movement using opportunistic individual identifications: application to sperm whales. Ecology 2001;82: 1417–1432. 10.1890/0012-9658(2001)082[1417:AOAMUO]2.0.CO;2.

[81] Garroway CJ, Bowman J, Wilson PJ. Complex social structure of southern flying squirrels is related to spatial proximity but not kinship. Behav Ecol Sociobiol. 2013;67: 113–122. 10.1007/s00265-012-1431-3.

[82] Mantel N. The detection of disease clustering and a generalized regression approach. Cancer Res. 1967;27: 209–220. 6018555

[83] Haccou P, Meelis E. Statistical analysis of behavioural data: an approach based on time-structured models New York: Oxford University Press; 1992.

[84] R Core Team. R: a language and environment for statistical computing Vienna: R Foundation for Statistical Computing 2016 Available from: https://www.r-project.org/.

[85] Dewey M. Metap: meta-analysis of significance values. R package version 0.6. 2014 Available from: http://cran.r-project.org/package=metap.

[86] Krause J, Krause S, Arlinghaus R, Psorakis I, Roberts S, Rutz C. Reality mining of animal social systems. Trends Ecol Evol. 2013;28: 541–551. 10.1016/j.tree.2013.06.002 23856617

[87] Rodgers TW, Giacalone J, Heske EJ, Janečka JE, Jansen PA, Phillips CA, et al Socio-spatial organization and kin structure in ocelots from integration of camera trapping and noninvasive genetics. J Mammal. 2015;96: 120–128. 10.1093/jmammal/gyu012.

[88] Wagner AP, Frank LG, Creel S. Spatial grouping in behaviourally solitary striped hyaenas, *Hyaena hyaena*. Anim Behav. 2008;75: 1131–1142. 10.1016/j.anbehav.2007.08.025.

[89] Baker PJ, Robertson CPJ, Funk SM, Harris S. Potential fitness benefits of group living in the red fox, *Vulpes vulpes*. Anim Behav. 1998;56: 1411–1424. 10.1006/anbe.1998.0950. 9933538

[90] van Ballenberghe V. Extraterritorial movements and dispersal of wolves in southcentral Alaska. J Mammal. 1983;64: 168–171. 10.2307/1380773

[91] Young AJ, Monfort SL. Stress and the costs of extra-territorial movement in a social carnivore. Biol Lett. 2009;5: 439–441. 10.1098/rsbl.2009.0032 19324630 PMC2781902

[92] Ilany A, Booms AS, Holekamp KE. Topological effects of network structure on long-term social network dynamics in a wild mammal. Ecol Lett. 2015;18: 687–695. 10.1111/ele.12447 25975663 PMC4486283

[93] McGregor HW, Legge S, Jones ME, Johnson CN. Extraterritorial hunting expeditions to intense fire scars by feral cats. Sci Rep. 2016;6: 22559 10.1038/srep22559 26932268 PMC4773836

[94] Baker PJ, Harris S. Interaction rates between members of a group of red foxes (*Vulpes vulpes*). Mamm Rev. 2000;30: 239–242. 10.1046/j.1365-2907.2000.00072.x.

[95] Mann J, Stanton MA, Patterson EM, Bienenstock EJ, Singh LO. Social networks reveal cultural behaviour in tool-using dolphins. Nat Commun. 2012;3: 980 10.1038/ncomms1983 22864573

[96] Mourier J, Vercelloni J, Planes S. Evidence of social communities in a spatially structured network of a free-ranging shark species. Anim Behav. 2012;83: 389–401. 10.1016/j.anbehav.2011.11.008.

[97] Shizuka D, Chaine AS, Anderson J, Johnson O, Laursen IM, Lyon BE. Across-year social stability shapes network structure in wintering migrant sparrows. Ecol Lett. 2014;17: 998–1007. 10.1111/ele.12304 24894316

[98] Louis M, Gally F, Barbraud C, Béesau J, Tixier P, Simon-Bouhet B, Le Rest K, Guinet C. Social structure and abundance of coastal bottlenose dolphins, *Tursiops truncatus*, in the Normano-Breton Gulf, English Channel. J Mammal. 2015;96: 481–493. 10.1093/jmammal/gyv053.

[99] Moorcroft PR. Mechanistic approaches to understanding and predicting mammalian space use: recent advances, future directions. J Mammal. 2012;93: 903–916. 10.1644/11-MAMM-S-254.1.

[100] Pinter-Wollman N, Isbell LA, Hart LA. The relationship between social behaviour and habitat familiarity in African elephants (*Loxodonta africana*). Proc R Soc B. 2009;276: 1009–1014. 10.1098/rspb.2008.1538 19129113 PMC2679069

[101] Carter AJ, Lee AEG, Marshall HH. Research questions should drive edge definitions in social network studies. Anim Behav. 2015;104: e7–e11. 10.1016/j.anbehav.2015.03.020.

[102] Palla G, Derényi I, Farkas I, Vicsek T. Uncovering the overlapping community structure of complex networks in nature and society. Nature. 2005;435: 814–818. 10.1038/nature03607 15944704

[103] Jacoby DMP, Busawon DS, Sims DW. Sex and social networking: the influence of male presence on social structure of female shark groups. Behav Ecol. 2010;21; 808–818. 10.1093/beheco/arq061.

[104] Blasi MF, Boitani L. Complex social structure of an endangered population of bottlenose dolphins (*Tursiops truncatus*) in the Aeolian Archipelago (Italy). PLoS One. 2014;9(12): e114849 10.1371/journal.pone.0114849 25494331 PMC4262461

[105] Palla G, Barabási A-L, Vicsek T. Quantifying social group evolution. Nature. 2007;446: 664–667. 10.1038/nature05670 17410175

[106] Lusseau D. The emergent properties of a dolphin social network. Proc R Soc B. 2003;270: S186–S188. 10.1098/rsbl.2003.0057 14667378 PMC1809954

[107] Kanngiesser P, Sueur C, Riedl K, Grossmann J, Call J. Grooming network cohesion and the role of individuals in a captive chimpanzee group. Am J Primat. 2011;73: 758–767. 10.1002/ajp.20914. 21698658

[108] Potts JR, Harris S, Giuggioli L. Territorial dynamics and stable home range formation for central place foragers. PLoS One. 2012;7(3): e34033 10.1371/journal.pone.0034033 22479510 PMC3316599

[109] Potts JR, Harris S, Giuggioli L. Quantifying behavioral changes in territorial animals caused by sudden population declines. Am Nat. 2013;182: E73–E82. 10.1086/671260 23933730

[110] Baird RW, Whitehead H. Social organization of mammal-eating killer whales: group stability and dispersal patterns. Can J Zool. 2000;78: 2096–2105. 10.1139/z00-155.

[111] Wey TW, Blumstein DT. Social cohesion in yellow-bellied marmots is established through age and kin structuring. Anim Behav. 2010;79: 1343–1352. 10.1016/j.anbehav.2010.03.008

[112] Carter KD, Brand R, Carter JK, Shorrocks B, Goldizen AW. Social networks, long-term associations and age-related sociability of wild giraffes. Anim Behav. 2013;86: 901–910. 10.1016/j.anbehav.2013.08.002.

[113] Hirsch BT, Prange S, Hauver SA, Gehrt SD. Raccoon social networks and the potential for disease transmission. PLoS One. 2013;8(10): e75830 10.1371/journal.pone.0075830 24130746 PMC3794951

[114] Christensen C, Radford AN. Dear enemies or nasty neighbors? Causes and consequences of variation in the responses of group-living species to territorial intrusions. Behav Ecol. 2018;29: 1004–1013. 10.1093/beheco/ary010

[115] White PCL, Harris S, Smith GC. Fox contact behaviour and rabies spread: a model for the estimation of contact probabilities between urban foxes at different population densities and its implications for rabies control in Britain. J Appl Ecol. 1995;32: 693–706. 10.2307/2404809.

[116] Hradsky BA, Kelly LT, Robley A, Wintle BA. FoxNet: An individual-based model framework to support management of an invasive predator, the red fox. J Appl Ecol. 2019; 10.1111/1365-2664.13374.

[117] Lowe S, Browne M, Boudjelas S, De Poorter. 100 of the world's worst invasive alien species: A selection from the global invasive species database. Gland, Switzerland: IUCN; 2000 Available from: www.issg.org/pdf/publications/worst_100/english_100_worst.pdf.

[118] Muller Z, Cantor M, Cuthill IC, Harris S. Giraffe social preferences are context-dependent. Anim Behav. 2018;146: 37–49. 10.1016/j.anbehav.2018.10.006.

