# Supplementary material for: Understanding the intricacy of canid social systems: Structure and temporal stability of red fox (Vulpes vulpes) groups
Source: PLoS One. 2019 Sep 11;14(9):e0220792. doi: 10.1371/journal.pone.0220792 (PMC6738593; doi:10.1371/journal.pone.0220792)
Supplement: S1 Appendix — Fig A. Distributions of sighting frequencies (days observed out of 40) for foxes > 5 months old. Distributions are plotted separately for each season and for all data pooled. Fig B. The proportion of dyads with simple ratio association indices (SRI) of increasing strength for individuals seen on ≥ 5 days in each season and territory. The proportion of individuals is on the y-axis and SRI, plotted between 0–1, is on the x-axis. Fig C. Proportions of individuals seen on ≥ 5 days in each territory and season with mean (blue) and maximum (yellow) simple ratio association indices (SRI) of increasing strength. Proportion of individuals is on the y-axis and SRI, plotted between 0–1, is on the x-axis. Fig D. The probability that pairs of foxes that associated on a given day would re-associate at a later time (lagged association rate, LAR) within communities 1–7, and the expected association rate if associations were random (null association rate, NAR) calculated across all data. Vertical lines show jack-knife standard errors. Fig E. The probability that pairs of foxes that associated on a given day would re-associate at a later time (lagged association rate, LAR) within each territory, and the expected association rate if associations were random (null association rate, NAR) calculated across all data. Vertical lines show jack-knife standard errors. (DOCX) [file pone.0220792.s001.docx]

S1 Appendix

## Supplementary figures


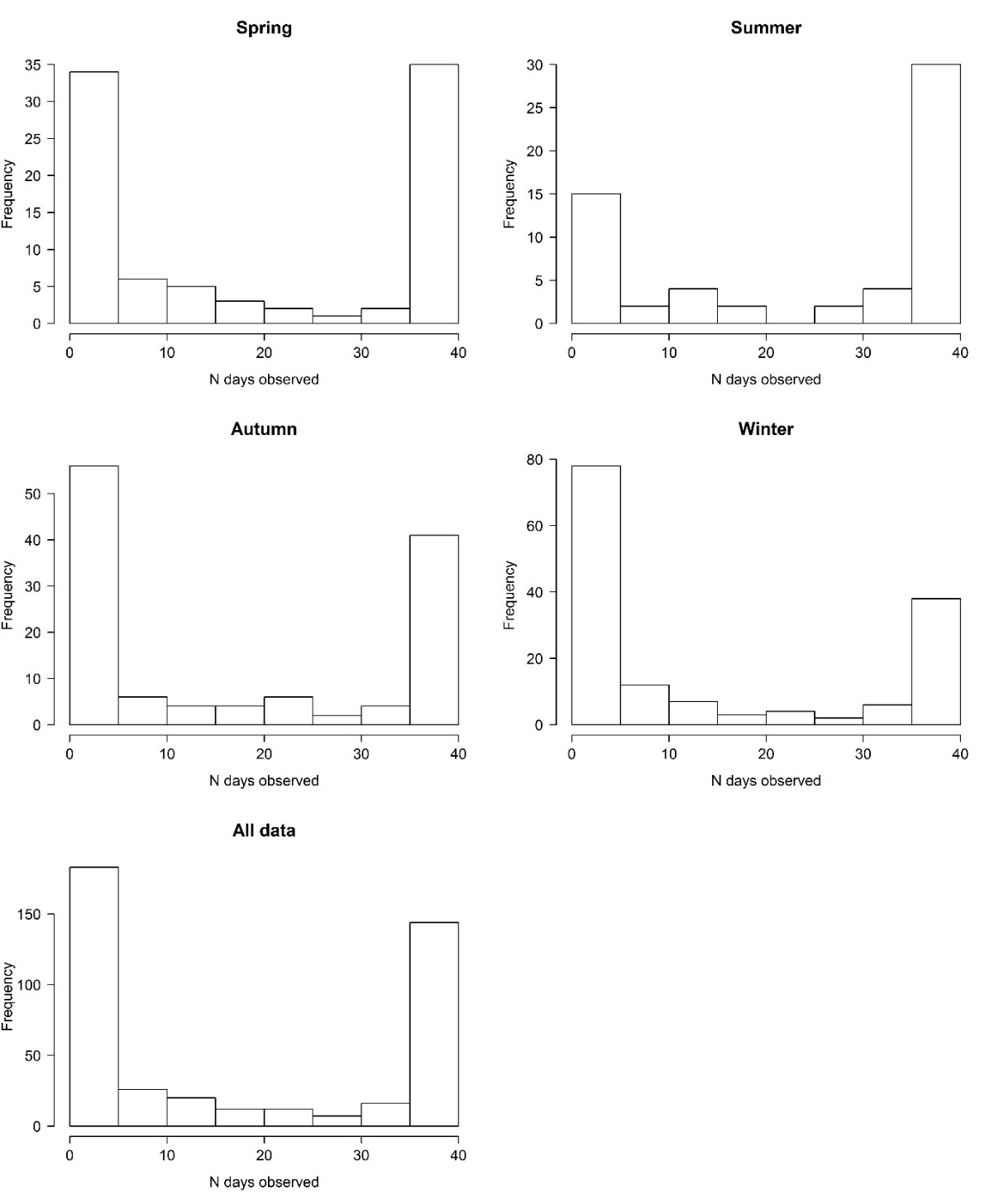


**Fig A. Distributions of sighting frequencies (days observed out of 40) for foxes > 5 months old.** Distributions are plotted separately for each season and for all data pooled.

|  | | **Territory 1** | | **Territory 2** | | **Territory 3** | | **Territory 4** | | **Territory 5** | | **Territory 6** | | **Territory 7** |
| --- | --- | --- | --- | --- | --- | --- | --- | --- | --- | --- | --- | --- | --- | --- |
| **Spring** | 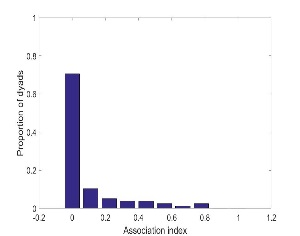 | | 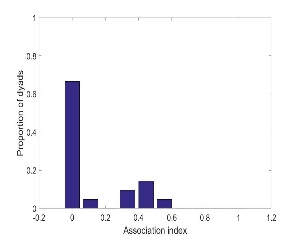 | | 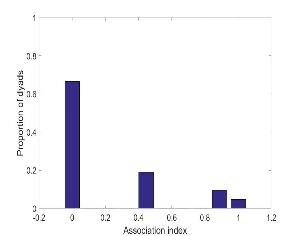 | | 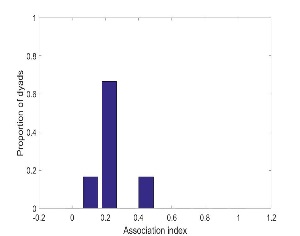 | | 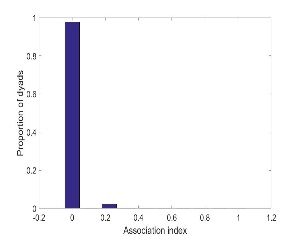 | | 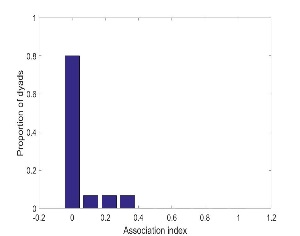 | | 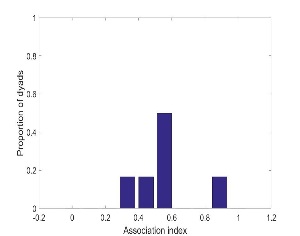 | |
| **Summer** | 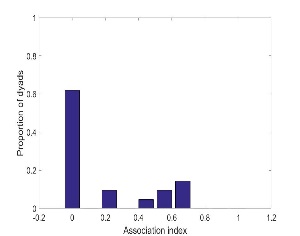 | | 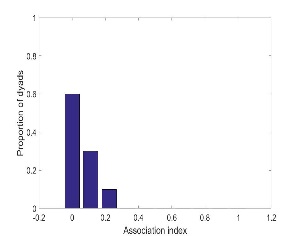 | | 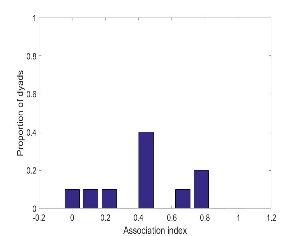 | | 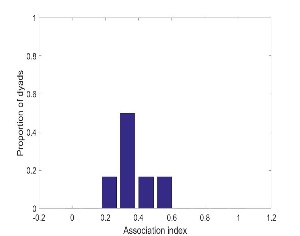 | | 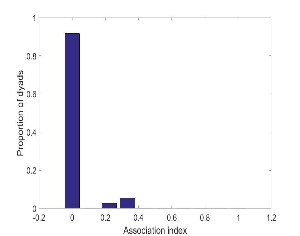 | | 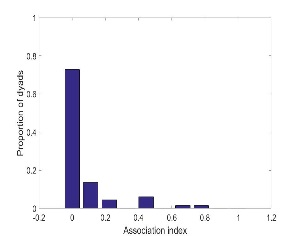 | | 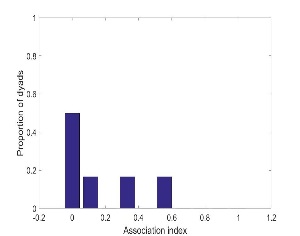 | |
| **Autumn** | 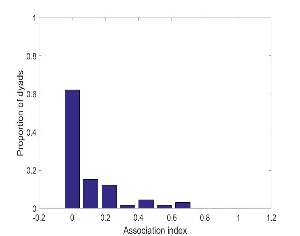 | | 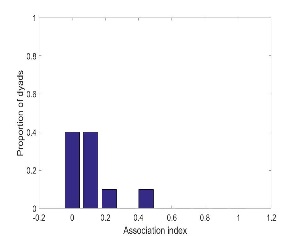 | | 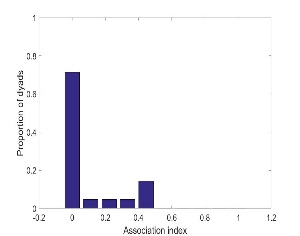 | | 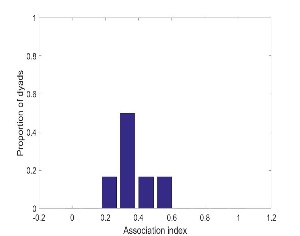 | | 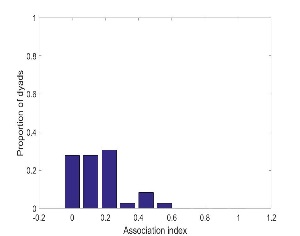 | | 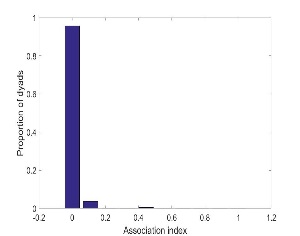 | | 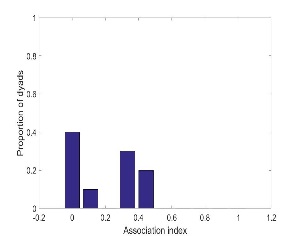 | |
| **Winter** | 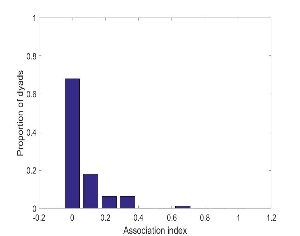 | | 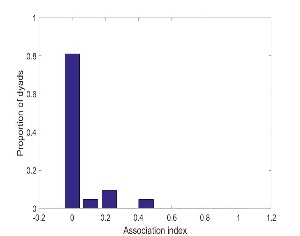 | | 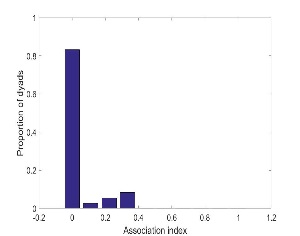 | | 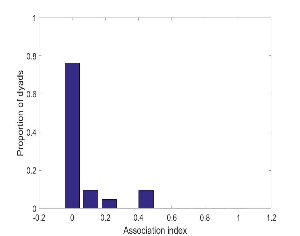 | | 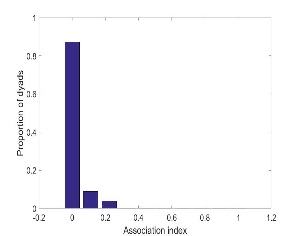 | | 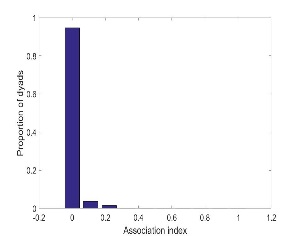 | | 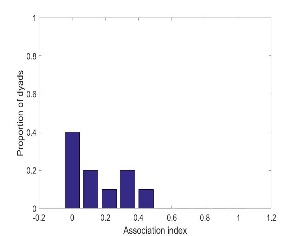 | |

**Fig B. The proportion of dyads with simple ratio association indices (SRI) of increasing strength for individuals seen on ≥ 5 days in each season and territory.** The proportion of individuals is on the y-axis and SRI, plotted between 0-1, is on the x-axis.

|  | **Territory 1** | **Territory 2** | **Territory 3** | **Territory 4** | **Territory 5** | **Territory 6** | **Territory 7** |
| --- | --- | --- | --- | --- | --- | --- | --- |
| **Spring** | 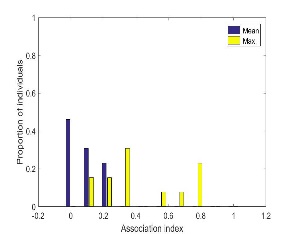 | 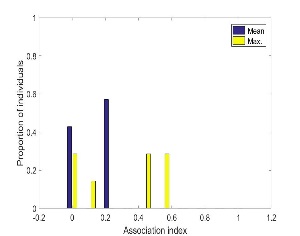 | 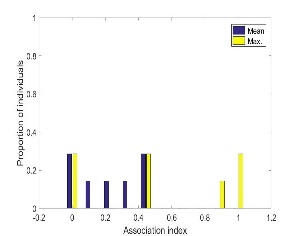 | 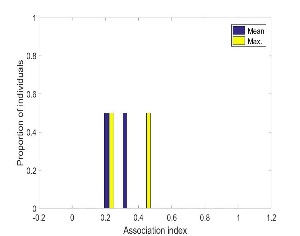 | 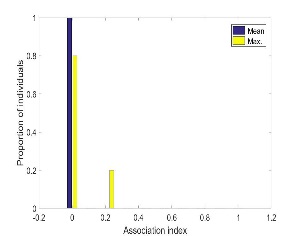 | 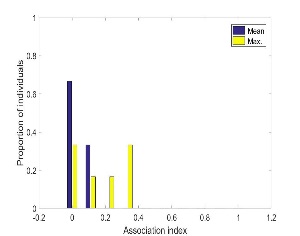 | 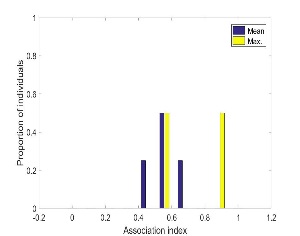 |
| **Summer** | 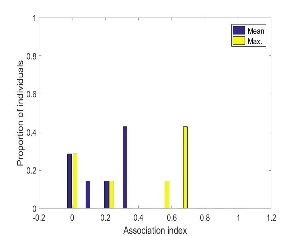 | 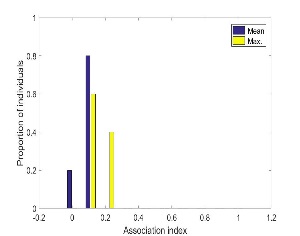 | 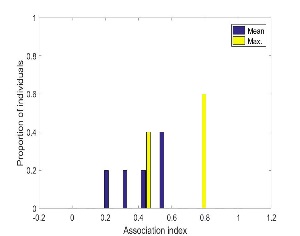 | 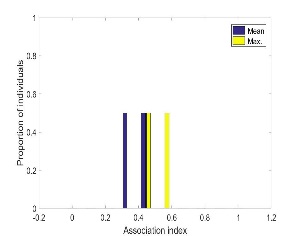 | 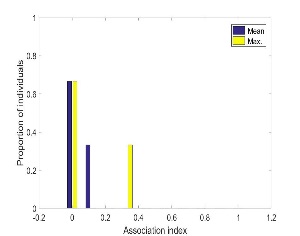 | 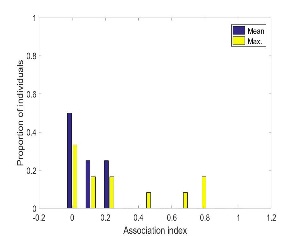 | 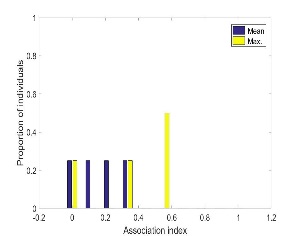 |
| **Autumn** | 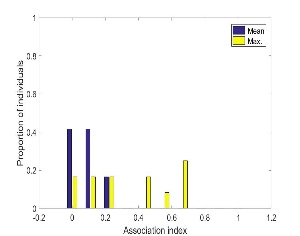 | 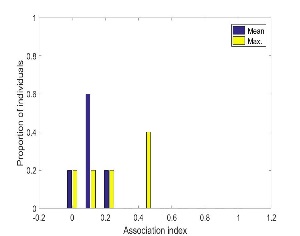 | 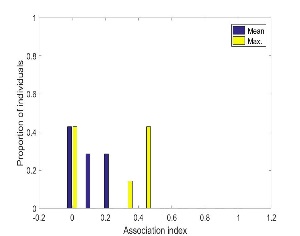 | 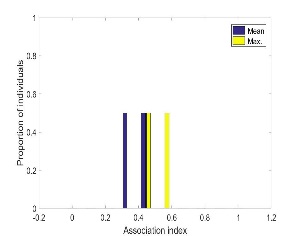 | 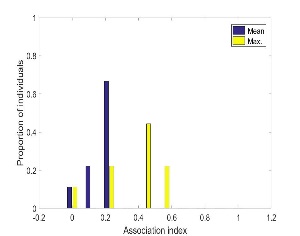 | 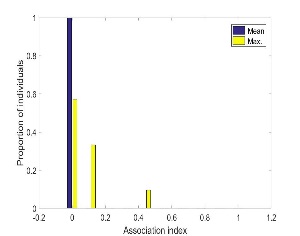 | 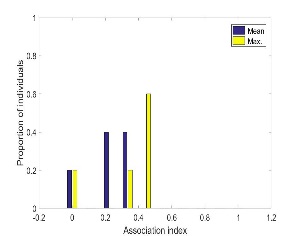 |
| **Winter** | 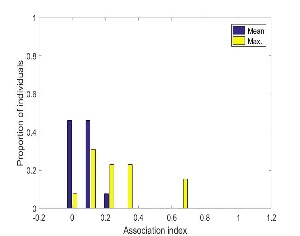 | 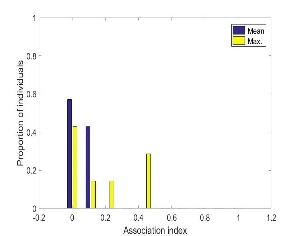 | 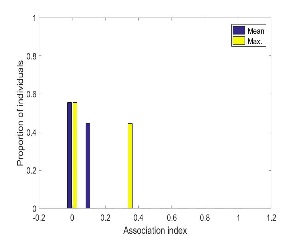 | 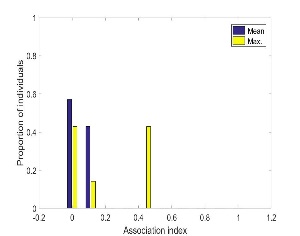 | 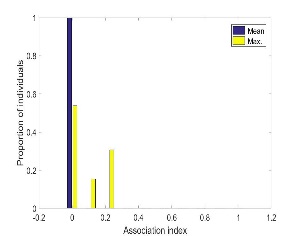 | 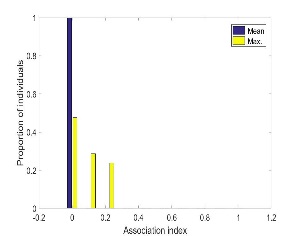 | 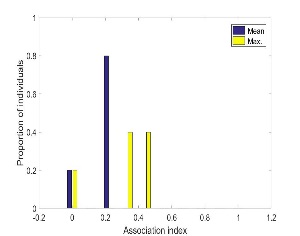 |

**Fig C. Proportions of individuals seen on ≥ 5 days in each territory and season with mean (blue) and maximum (yellow) simple ratio association indices (SRI) of increasing strength.** Proportion of individuals is on the y-axis and SRI, plotted between 0-1, is on the x-axis.


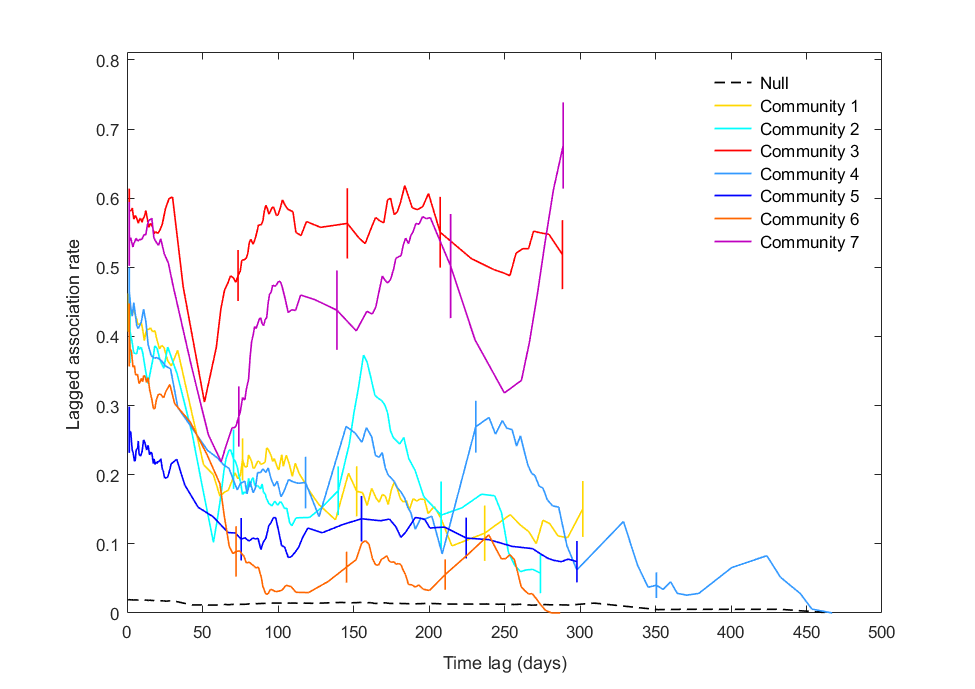


**Fig D. The probability that pairs of foxes that associated on a given day would re-associate at a later time (lagged association rate, LAR) within communities 1-7, and the expected association rate if associations were random (null association rate, NAR) calculated across all data.** Vertical lines show jack-knife standard errors.


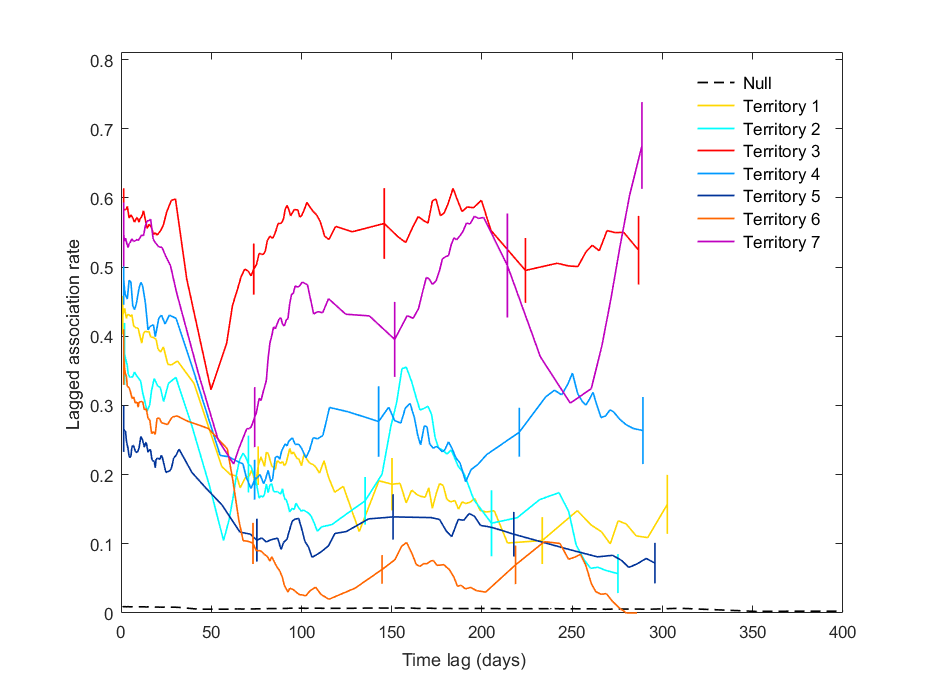


**Fig E. The probability that pairs of foxes that associated on a given day would re-associate at a later time (lagged association rate, LAR) within each territory, and the expected association rate if associations were random (null association rate, NAR) calculated across all data.** Vertical lines show jack-knife standard errors.
