# Supplementary material for: Understanding the intricacy of canid social systems: Structure and temporal stability of red fox (Vulpes vulpes) groups
Source: PLoS One. 2019 Sep 11;14(9):e0220792. doi: 10.1371/journal.pone.0220792 (PMC6738593; doi:10.1371/journal.pone.0220792)
Supplement: S2 Appendix — Table A. Summary of survey effort and association data used to construct social networks. T = territory; days = sampling periods; patches = camera sites; observations = observations of filtered individuals; true associations = dyadic associations rather than self-associations; S = maximum likelihood estimate of social differentiation; r = correlation between true and estimated association indices; SE = standard error. Bold values indicate S ≥ 0.2 or r ≥ 0.4. *—some patches were not used for all four seasons. Table B. Results of the Manly/Bejder tests for preferred and avoided companions for the combined dataset and separately for each territory and season network. Dashes indicate degenerate networks that were too sparse to permute. * indicates the p-value was close to significance (0.1 > p > 0.05). Table C. Parameter estimates and interpretation of the best-fitting exponential decay models fitted to lagged association rates (LARs) for the whole dataset and separate seasons, communities and territories. Standard errors were estimated by jack-knifing over one day. The models with the lowest QAIC each contained proportions of rapid disassociations (RD), preferred companions (PC) and casual acquaintances (CA) of one or two types, defined in the formulae as a1-a4. Lag = time lag in days. The top two models are presented for autumn as ΔQAIC < 2. AR indicates the probability of re-association after given time lags in days. Table D. Mantel test correlations (R) between seasonal association matrices with the mean p-value and standard deviation (SD) calculated from three runs each with 10,000 permutations. Season pair denotes the two surveys compared, listed in the order of data collection: spring (SP), summer (SU), autumn (AU) and winter (WI). Time between seasons indicates how close in time the compared surveys were: 1 = consecutive, 2 = gap of one season and 3 = gap of 2 seasons. Significant p-values (p < 0.05) and those close to significance (0.1 > p > 0.05, marked with * [file pone.0220792.s002.docx]

S2 Appendix

## Supplementary tables

**Table A. Summary of survey effort and association data used to construct social networks.** T = territory; days = sampling periods; patches = camera sites; observations = observations of filtered individuals; true associations = dyadic associations rather than self-associations; *S* = maximum likelihood estimate of social differentiation; *r* = correlation between true and estimated association indices; SE = standard error. Bold values indicate *S* ≥ 0.2 or *r* ≥ 0.4. * - denotes the total number of unique dates when any survey occurred; + - some patches were not used for all four seasons.

| **T** | **Season** | **Total *N* of survey days** | **Total *N* of patches used in survey** | **Total *N* of foxes observed before filtering** | **Total *N* of foxes observed on ≥ 5 days** | **Total *N* of observations recorded** | **Total *N* of true associations recorded** | **Mean *N* observations recorded per day** | **Mean *N* foxes observed per day** | **Mean *N* days when each dyad associated** | **Mean *N true* associations recorded per fox** | ***S*** | **SE*_S_*** | ***r*** | **SE*_r_*** |
| --- | --- | --- | --- | --- | --- | --- | --- | --- | --- | --- | --- | --- | --- | --- | --- |
| All | All | 629* | 36+ | 174 | 83 | 38,273 | 3914 | 60.8 | 11.5 | 0.9 | 76.3 | **1.255** | 0.012 | 0.197 | 0.003 |
| T1 | Spring | 40 | 4 | 15 | 13 | 1623 | 360 | 40.6 | 12.5 | 4.0 | 48.0 | **1.377** | 0.010 | **0.725** | 0.016 |
|  | Summer | 40 | 4 | 7 | 7 | 941 | 217 | 23.5 | 6.5 | 8.1 | 48.6 | **1.199** | 0.029 | **0.877** | 0.024 |
|  | Autumn | 40 | 4 | 15 | 12 | 1816 | 304 | 45.4 | 10.6 | 4.1 | 45.3 | **1.262** | 0.019 | **0.793** | 0.022 |
|  | Winter | 40 | 4 | 21 | 13 | 1896 | 242 | 47.4 | 12.6 | 2.7 | 32.6 | **1.272** | 0.020 | **0.741** | 0.029 |
| T2 | Spring | 40 | 4 | 11 | 7 | 1425 | 138 | 35.6 | 5.1 | 5.2 | 31.4 | **1.271** | 0.028 | **0.846** | 0.018 |
|  | Summer | 40 | 4 | 6 | 5 | 941 | 26 | 23.5 | 4.8 | 2.6 | 10.4 | **0.787** | 0.116 | **0.833** | 0.092 |
|  | Autumn | 40 | 4 | 8 | 5 | 1292 | 58 | 32.3 | 4.2 | 4.4 | 17.6 | **1.032** | 0.063 | **0.866** | 0.059 |
|  | Winter | 40 | 4 | 20 | 7 | 1197 | 52 | 29.9 | 4.9 | 2.1 | 12.6 | **1.298** | 0.046 | **0.627** | 0.043 |
| T3 | Spring | 40 | 4 | 12 | 7 | 1392 | 257 | 34.8 | 5.4 | 8.6 | 51.7 | **1.216** | 0.013 | **0.784** | 0.021 |
|  | Summer | 40 | 4 | 8 | 5 | 882 | 207 | 22.1 | 4.8 | 16.4 | 65.6 | **0.623** | 0.049 | **1.007** | 0.037 |
|  | Autumn | 40 | 4 | 17 | 7 | 1186 | 109 | 29.7 | 5.3 | 4.1 | 24.9 | **1.349** | 0.019 | **0.798** | 0.022 |
|  | Winter | 40 | 4 | 20 | 9 | 1156 | 89 | 28.9 | 5.1 | 1.9 | 15.1 | **1.347** | 0.033 | **0.623** | 0.028 |
| T4 | Spring | 40 | 4 | 6 | 4 | 1033 | 77 | 25.8 | 3.9 | 10.2 | 30.5 | **0.296** | 0.119 | **0.646** | 0.142 |
|  | Summer | 40 | 4 | 4 | 4 | 901 | 110 | 22.5 | 4.0 | 15.3 | 46.0 | **0.233** | 0.104 | **0.697** | 0.155 |
|  | Autumn | 40 | 4 | 16 | 10 | 1719 | 321 | 43.0 | 7.9 | 4.9 | 44.4 | **1.400** | 0.008 | **0.762** | 0.013 |
|  | Winter | 40 | 4 | 17 | 7 | 1026 | 68 | 25.7 | 4.7 | 2.6 | 15.7 | **1.358** | 0.029 | **0.684** | 0.033 |
| T5 | Spring | 40 | 5 | 20 | 10 | 1050 | 14 | 26.3 | 6.0 | 0.3 | 2.6 | **0.831** | 0.103 | 0.215 | 0.036 |
|  | Summer | 40 | 5 | 13 | 9 | 930 | 47 | 23.3 | 4.8 | 1.1 | 8.7 | **1.341** | 0.048 | **0.420** | 0.017 |
|  | Autumn | 40 | 5 | 24 | 9 | 1763 | 264 | 44.1 | 8.2 | 6.5 | 52.0 | **0.866** | 0.045 | **0.978** | 0.032 |
|  | Winter | 40 | 5 | 25 | 13 | 1436 | 70 | 35.9 | 7.8 | 0.8 | 9.7 | **1.072** | 0.067 | **0.478** | 0.043 |
| T6 | Spring | 40 | 4 | 16 | 6 | 786 | 41 | 19.7 | 4.3 | 1.9 | 9.7 | **1.232** | 0.063 | **0.653** | 0.075 |
|  | Summer | 40 | 4 | 14 | 12 | 1601 | 247 | 40.0 | 8.9 | 3.1 | 34.2 | **1.391** | 0.016 | **0.658** | 0.025 |
|  | Autumn | 40 | 4 | 31 | 21 | 1091 | 67 | 27.3 | 12.2 | 0.3 | 6.2 | **0.886** | 0.054 | 0.194 | 0.023 |
|  | Winter | 40 | 4 | 30 | 21 | 1497 | 91 | 37.4 | 12.3 | 0.4 | 7.5 | **0.964** | 0.067 | 0.250 | 0.022 |
| T7 | Spring | 40 | 4 | 5 | 4 | 1203 | 225 | 30.1 | 4.0 | 22.2 | 66.5 | **0.312** | 0.067 | **0.856** | 0.045 |
|  | Summer | 40 | 4 | 6 | 4 | 692 | 46 | 17.3 | 3.4 | 6.7 | 20.0 | **1.166** | 0.061 | **0.807** | 0.039 |
|  | Autumn | 40 | 4 | 11 | 5 | 886 | 93 | 22.2 | 4.4 | 8.1 | 32.4 | **1.068** | 0.018 | **1.100** | 0.041 |
|  | Winter | 40 | 4 | 17 | 5 | 952 | 69 | 23.8 | 4.1 | 6.2 | 24.8 | **1.073** | 0.020 | **1.074** | 0.059 |

**Table B. Results of the Manly/Bejder tests for preferred and avoided companions for the combined dataset and separately for each territory and season network.** Dashes indicate degenerate networks that were too sparse to permute. * indicates the *p*-value was close to significance (0.1 > *p* > 0.05).

| **Territory** | **Season** | **Long-term ^a^** | | | **Short-term ^b^** | | | **Long-term ^b^** | | | **Individual differences in gregariousness ^b^** | | |
| --- | --- | --- | --- | --- | --- | --- | --- | --- | --- | --- | --- | --- | --- |
|  |  | **CV*_obs_*** | **CV*_rand_*** | ***p*** | **Mean*_obs_*** | **Mean*_rand_*** | ***p*** | **CV*_obs_*** | **CV*_rand_*** | ***p*** | **SD*_obs_*** | **SD*_rand_*** | ***p*** |
| All | All | 6.355 | 4.625 | **<0.001** | 0.008 | 0.012 | **<0.001** | 5.565 | 4.033 | **<0.001** | 0.163 | 0.129 | **<0.001** |
| 1 | Spring | 1.893 | 1.445 | **<0.001** | 0.100 | 0.107 | **0.001** | 1.893 | 0.739 | **<0.001** | 0.208 | 0.045 | **<0.001** |
| 1 | Summer | 1.350 | 1.337 | **0.037** | 0.202 | 0.214 | **0.013** | 1.350 | 0.694 | **<0.001** | 0.214 | 0.041 | **<0.001** |
| 1 | Autumn | 1.585 | 1.375 | **<0.001** | 0.151 | 0.153 | 0.187 | 1.182 | 0.522 | **<0.001** | 0.128 | 0.032 | **<0.001** |
| 1 | Winter | 1.712 | 1.426 | **<0.001** | 0.068 | 0.074 | **0.003** | 1.712 | 0.654 | **<0.001** | 0.155 | 0.034 | **<0.001** |
| 2 | Spring | 1.485 | 1.491 | 0.809 | 0.131 | 0.125 | 0.931 | 1.485 | 1.387 | **0.073*** | 0.080 | 0.063 | 0.210 |
| 2 | Summer | 0.920 | 0.742 | **0.013** | 0.065 | 0.060 | 0.967 | 0.920 | 0.780 | 0.200 | 0.022 | 0.016 | 0.122 |
| 2 | Autumn | 1.160 | 1.129 | **0.084*** | 0.111 | 0.118 | 0.127 | 1.160 | 0.957 | **0.022** | 0.048 | 0.039 | 0.179 |
| 2 | Winter | 2.045 | 1.977 | **0.043** | 0.074 | 0.075 | 0.409 | 1.650 | 1.307 | **0.010** | 0.035 | 0.033 | 0.484 |
| 3 | Spring | 1.532 | 1.506 | **0.002** | 0.302 | 0.289 | 0.941 | 1.182 | 1.004 | **<0.001** | 0.103 | 0.066 | 0.107 |
| 3 | Summer | 0.612 | 0.580 | **0.001** | 0.410 | 0.387 | 0.985 | 0.612 | 0.517 | **0.021** | 0.101 | 0.038 | **0.001** |
| 3 | Autumn | 1.669 | 1.671 | 0.471 | 0.104 | 0.109 | **0.057*** | 1.669 | 1.286 | **<0.001** | 0.090 | 0.049 | **0.033** |
| 3 | Winter | 2.146 | 2.118 | **0.048** | 0.116 | 0.119 | 0.221 | 1.164 | 0.061 | **0.056*** | 0.045 | 0.038 | 0.266 |
| 4 | Spring | 0.438 | 0.411 | **0.085*** | 0.254 | 0.262 | 0.265 | 0.438 | 0.376 | 0.250 | 0.025 | 0.017 | 0.149 |
| 4 | Summer | 0.319 | 0.216 | **0.026** | 0.383 | 0.364 | 0.917 | 0.319 | 0.333 | 0.567 | 0.078 | 0.034 | **<0.001** |
| 4 | Autumn | 1.828 | 1.812 | **0.019** | 0.198 | 0.217 | **0.003** | 1.307 | 0.869 | **<0.001** | 0.181 | 0.048 | **<0.001** |
| 4 | Winter | 1.961 | 1.959 | 0.295 | 0.138 | 0.146 | **0.055*** | 1.154 | 0.903 | **0.002** | 0.051 | 0.031 | **0.079*** |
| 5 | Spring | - | - | - | 0.033 | 0.034 | 0.318 | 1.616 | 1.156 | **0.034** | 0.009 | 0.016 | 0.757 |
| 5 | Summer | - | - | - | 0.098 | 0.105 | **0.041** | 1.462 | 1.119 | **0.005** | 0.047 | 0.039 | 0.295 |
| 5 | Autumn | 0.879 | 0.742 | **<0.001** | 0.163 | 0.160 | 0.741 | 0.879 | 0.703 | **<0.001** | 0.094 | 0.040 | **0.002** |
| 5 | Winter | 2.236 | 2.148 | **0.068*** | 0.035 | 0.036 | 0.357 | 1.571 | 1.496 | 0.231 | 0.052 | 0.057 | 0.562 |
| 6 | Spring | - | - | - | 0.122 | 0.140 | **0.013** | 0.893 | 0.429 | **<0.001** | 0.042 | 0.028 | 0.104 |
| 6 | Summer | 2.107 | 2.051 | **0.004** | 0.142 | 0.146 | 0.114 | 1.406 | 0.842 | **<0.001** | 0.146 | 0.043 | **<0.001** |
| 6 | Autumn | 4.562 | 3.724 | **<0.001** | 0.022 | 0.022 | 0.438 | 2.670 | 1.199 | **<0.001** | 0.111 | 0.047 | **0.002** |
| 6 | Winter | 3.856 | 2.934 | **<0.001** | 0.017 | 0.019 | **0.001** | 2.843 | 1.649 | **<0.001** | 0.075 | 0.054 | **0.021** |
| 7 | Spring | 0.348 | 0.338 | 0.188 | 0.554 | 0.563 | 0.269 | 0.348 | 0.151 | **<0.001** | 0.104 | 0.030 | **<0.001** |
| 7 | Summer | - | - | - | 0.167 | 0.161 | 0.783 | 1.377 | 0.985 | **<0.001** | 0.047 | 0.036 | 0.225 |
| 7 | Autumn | 0.945 | 0.944 | 0.265 | 0.338 | 0.310 | 0.985 | 0.345 | 0.223 | **0.044** | 0.056 | 0.026 | **0.004** |
| 7 | Winter | 0.973 | 0.962 | 0.168 | 0.258 | 0.239 | 0.982 | 0.390 | 0.245 | **0.041** | 0.027 | 0.016 | **0.064*** |

^a^ Associations were permuted within sampling periods (days) and isolates were included; ^b^ Groups were permuted within days and isolates were excluded; *_obs_* = observed data; *_rand_* = permuted data.

**Table C. Parameter estimates and interpretation of the best-fitting exponential decay models fitted to lagged association rates (LARs) for the whole dataset and separate seasons, communities and territories.** Standard errors were estimated by jack-knifing over one day. The models with the lowest QAIC each contained proportions of rapid disassociations (RD), preferred companions (PC) and casual acquaintances (CA) of one or two types, defined in the formulae as a1-a4. Lag = time lag in days. The top two models are presented for autumn as ∆QAIC < 2. AR indicates the probability of re-association after given time lags in days.

| **Data** | **Model description**  **and formula** | **Parameter** | **Estimate** | **SE** | **Model component** | **Value ^a^ (SE range)** | **AR at lag = 1** | **AR at**  **lag = 365** |
| --- | --- | --- | --- | --- | --- | --- | --- | --- |
| All data | RD & two levels of CA:  $a3\times exp(-a1\times lag)+ a4\times exp(-a2\times lag)$ | a1 | 0.04993 | 0.007 | Duration of CA type 1 | 20 days (17.6-23.3) | 0.46 | 0.15 |
|  |  | a2 | 0.002254 | 0.0003 | Duration of CA type 2 | 443.6 days (391.5-511.7) |  |  |
|  |  | a3 | 0.13655 | 0.022 | Proportion of CA type 1 | 13.7 % (11.5-15.9) |  |  |
|  |  | a4 | 0.33117 | 0.021 | Proportion of CA type 2 | 33.1 % (31-35.2) |  |  |
|  |  |  |  |  | Proportion of RD | 53.2 % (48.9-57.5) |  |  |
| Spring ^b^ | RD & CA:  $a2\times exp(-a1\times lag)$ | a1 | 0.005204 | 0.002 | Duration of CA | 192.2 days (138.8-312.1) | 0.55 |  |
|  |  | a2 | 0.5476 | 0.023 | Proportion of CA | 54.8 % (52.5-57.1) |  |  |
|  |  |  |  |  | Proportion of RD | 45.2 % (42.9-47.5) |  |  |
| Summer ^b^ | RD & CA:  $a2\times exp(-a1\times lag)$ | a1 | 0.007118 | 0.002 | Duration of CA | 140.5 days (109.7-195.4) | 0.49 |  |
|  |  | a2 | 0.49069 | 0.025 | Proportion of CA | 49.1 % (46.6-51.6) |  |  |
|  |  |  |  |  | Proportion of RD | 50.9 % (48.4-53.4) |  |  |
| Autumn ^b^ (top two models) | RD & CA (QAIC = 48392.1):  $a2\times exp(-a1\times lag)$ | a1 | 0.004085 | 0.003 | Duration of CA | 244.8 days (135.6-1256.1) | 0.4 |  |
|  |  | a2 | 0.39753 | 0.026 | Proportion of CA | 39.8 % (37.1-42.4) |  |  |
|  |  |  |  |  | Proportion of RD | 60.3 % (57.6-62.9) |  |  |
|  | RD, PC & CA (QAIC = 48392.7):  $a2+a3\times exp(-a1\times lag)$ | a1 | 0.041737 | 0.079 | Duration of CA | 24 days (8.3--26.6) | 0.4 |  |
|  |  | a2 | 0.33525 | 0.095 | Proportion of PC | 33.5 % (24-43) |  |  |
|  |  | a3 | 0.067655 | 0.093 | Proportion of CA | 6.8 % (-2.6-16.1) |  |  |
|  |  |  |  |  | Proportion of RD | 59.7 % (40.9-78.5) |  |  |
| Winter ^b^ | RD & two levels of CA:  $a3\times exp(-a1\times lag)+ a4\times exp(-a2\times lag)$ | a1 | 0.26667 | 0.197 | Duration of CA type 1 | 3.8 days (2.2-14.3) | 0.34 |  |
|  |  | a2 | 0.016644 | 0.01 | Duration of CA type 2 | 60.1 days (37.1-157.3) |  |  |
|  |  | a3 | 0.079958 | 0.066 | Proportion of CA type 1 | 8 % (1.4-14.6) |  |  |
|  |  | a4 | 0.28013 | 0.057 | Proportion of CA type 2 | 28 % (22.4-33.7) |  |  |
|  |  |  |  |  | Proportion of RD | 64 % (51.7-76.3) |  |  |
| Community 1 | RD, PC & CA:  $a2+a3\times exp(-a1\times lag)$ | a1 | 0.01633 | 0.004 | Duration of CA | 61.2 days (49.2-81.1) | 0.46 | 0.14 |
|  |  | a2 | 0.13606 | 0.022 | Proportion of PC | 13.6 % (11.4-15.8) |  |  |
|  |  | a3 | 0.32552 | 0.031 | Proportion of CA | 32.6 % (29.5-35.7) |  |  |
|  |  |  |  |  | Proportion of RD | 53.8 % (48.5-59.1) |  |  |
| Territory 1 | RD, PC & CA:  $a2+a3\times exp(-a1\times lag)$ | a1 | 0.01514 | 0.004 | Duration of CA | 66.1 days (52.2-89.8) | 0.45 | 0.13 |
|  |  | a2 | 0.13257 | 0.023 | Proportion of PC | 13.3 % (11-15.6) |  |  |
|  |  | a3 | 0.32083 | 0.031 | Proportion of CA | 32.1 % (29-35.2) |  |  |
|  |  |  |  |  | Proportion of RD | 54.7 % (49.3-60.1) |  |  |
| Community 2 | RD, PC & CA:  $a2+a3\times exp(-a1\times lag)$ | a1 | 0.03535 | 0.008 | Duration of CA | 28.3 days (23.1-36.6) | 0.44 | 0.18 |
|  |  | a2 | 0.18161 | 0.024 | Proportion of PC | 18.2 % (15.8-20.6) |  |  |
|  |  | a3 | 0.26981 | 0.053 | Proportion of CA | 27 % (21.7-32.3) |  |  |
|  |  |  |  |  | Proportion of RD | 54.9 % (47.2-62.6) |  |  |
| Territory 2 | RD, PC & CA:  $a2+a3\times exp(-a1\times lag)$ | a1 | 0.0359 | 0.008 | Duration of CA | 27.9 days (22.8-35.8) | 0.4 | 0.18 |
|  |  | a2 | 0.1754 | 0.023 | Proportion of PC | 17.5 % (15.2-19.8) |  |  |
|  |  | a3 | 0.22929 | 0.05 | Proportion of CA | 22.9 % (17.9-27.9) |  |  |
|  |  |  |  |  | Proportion of RD | 59.5 % (52.2-66.8) |  |  |
| Community 3 | RD, PC & CA:  $a2+a3\times exp(-a1\times lag)$ | a1 | 0.08104 | 0.048 | Duration of CA | 12.3 days (7.7-30.5) | 0.59 | 0.55 |
|  |  | a2 | 0.54552 | 0.023 | Proportion of PC | 54.6 % (52.3-56.9) |  |  |
|  |  | a3 | 0.04708 | 0.032 | Proportion of CA | 4.7 % (1.5-7.9) |  |  |
|  |  |  |  |  | Proportion of RD | 40.7 % (35.2-46.2) |  |  |
| Territory 3 | RD, PC & CA:  $a2+a3\times exp(-a1\times lag)$ | a1 | 0.084102 | 0.057 | Duration of CA | 11.9 days (7.1-36.6) | 0.55 | 0.54 |
|  |  | a2 | 0.5457 | 0.023 | Proportion of PC | 54.6 % (52.3-56.8) |  |  |
|  |  | a3 | 0.047914 | 0.032 | Proportion of CA | 4.8 % (1.6-8) |  |  |
|  |  |  |  |  | Proportion of RD | 40.6 % (35.2-46.1) |  |  |
| Community 4 | RD & two levels of CA:  $a3\times exp(-a1\times lag)+ a4\times exp(-a2\times lag)$ | a1 | 0.002869 | 0.0005 | Duration of CA type 1 | 348.6 days (301.2-413.7) | 0.49 | 0.1 |
|  |  | a2 | 0.054138 | 0.008 | Duration of CA type 2 | 18.5 days (21.6-16.2) |  |  |
|  |  | a3 | 0.29202 | 0.024 | Proportion of CA type 1 | 29.2 % (26.8-31.6) |  |  |
|  |  | a4 | 0.20848 | 0.048 | Proportion of CA type 2 | 20.8 % (16-25.7) |  |  |
|  |  |  |  |  | Proportion of RD | 50 % (42.7-57.2) |  |  |
| Territory 4 | RD & two levels of CA:  $a3\times exp(-a1\times lag)+ a4\times exp(-a2\times lag)$ | a1 | -0.00205 | 0.001 | Duration of CA type 1 | 488.1 days (349.7-807.6) | 0.52 | 0.38 |
|  |  | a2 | 0.028274 | 0.005 | Duration of CA type 2 | 35.4 days (29.7-43.7) |  |  |
|  |  | a3 | 0.17175 | 0.033 | Proportion of CA type 1 | 17.2 % (13.8-20.5) |  |  |
|  |  | a4 | 0.35943 | 0.06 | Proportion of CA type 2 | 35.9 % (30-41.9) |  |  |
|  |  |  |  |  | Proportion of RD | 46.9 % (37.6-56.2) |  |  |
| Community 5 | RD, PC & CA:  $a2+a3\times exp(-a1\times lag)$ | a1 | 0.02255 | 0.009 | Duration of CA | 44.4 days (31.7-73.8) | 0.27 | 0.1 |
|  |  | a2 | 0.09952 | 0.02 | Proportion of PC | 10 % (8-12) |  |  |
|  |  | a3 | 0.16982 | 0.035 | Proportion of CA | 17 % (13.5-20.5) |  |  |
|  |  |  |  |  | Proportion of RD | 73.1 % (67.6-78.6) |  |  |
| Territory 5 | RD, PC & CA:  $a2+a3\times exp(-a1\times lag)$ | a1 | 0.02123 | 0.008 | Duration of CA | 47.1 days (34.2-75.6) | 0.27 | 0.1 |
|  |  | a2 | 0.09859 | 0.02 | Proportion of PC | 9.9 % (7.9-11.9) |  |  |
|  |  | a3 | 0.17457 | 0.035 | Proportion of CA | 17.5 % (14-21) |  |  |
|  |  |  |  |  | Proportion of RD | 72.7 % (67.2-78.2) |  |  |
| Community 6 | RD, PC & CA:  $a2+a3\times exp(-a1\times lag)$ | a1 | 0.02709 | 0.004 | Duration of CA | 36.9 days (32.2-43.3) | 0.43 | 0.05 |
|  |  | a2 | 0.04879 | 0.011 | Proportion of PC | 4.9 % (3.8-6) |  |  |
|  |  | a3 | 0.38864 | 0.059 | Proportion of CA | 38.9 % (33-44.8) |  |  |
|  |  |  |  |  | Proportion of RD | 56.3 % (49.3-63.3) |  |  |
| Territory 6 | RD, PC & CA:  $a2+a3\times exp(-a1\times lag)$ | a1 | 0.02473 | 0.004 | Duration of CA | 40.4 days (34.8-48.2) | 0.38 | 0.05 |
|  |  | a2 | 0.04565 | 0.011 | Proportion of PC | 4.6 % (3.5-5.7) |  |  |
|  |  | a3 | 0.34244 | 0.054 | Proportion of CA | 34.2 % (28.8-39.6) |  |  |
|  |  |  |  |  | Proportion of RD | 61.2 % (54.7-67.7) |  |  |
| Community 7 | RD & two levels of CA:  $a3\times exp(-a1\times lag)+ a4\times exp(-a2\times lag)$ | a1 | -0.002 | 0.0007 | Duration of CA type 1 | 500.6 days (377.6-742.5) | 0.58 | 0.45 |
|  |  | a2 | 0.037769 | 0.005 | Duration of CA type 2 | 26.5 days (31-23.1) |  |  |
|  |  | a3 | 0.32896 | 0.044 | Proportion of CA type 1 | 32.9 % (28.5-37.3) |  |  |
|  |  | a4 | 0.27456 | 0.057 | Proportion of CA type 2 | 27.5 % (21.7-33.2) |  |  |
|  |  |  |  |  | Proportion of RD | 39.6 % (29.5-49.8) |  |  |
| Territory 7 | RD & two levels of CA:  $a3\times exp(-a1\times lag)+ a4\times exp(-a2\times lag)$ | a1 | 0.038138 | 0.006 | Duration of CA type 1 | 26.2 days (22.9-30.7) | 0.59 | 0.68 |
|  |  | a2 | -0.002 | 0.001 | Duration of CA type 2 | 500 days (377.1-741.6) |  |  |
|  |  | a3 | 0.27641 | 0.057 | Proportion of CA type 1 | 27.6 % (21.9-33.4) |  |  |
|  |  | a4 | 0.32672 | 0.044 | Proportion of CA type 2 | 32.7 % (28.3-37) |  |  |
|  |  |  |  |  | Proportion of RD | 39.7 % (29.6-49.8) |  |  |

^a^ Durations were calculated by $1/a1$ or $1/a2$. Proportions of RD were calculated by $1-(a2+a3)$ for *RD, PC & CA* models or $1-(a3+a4)$for *RD & two levels of CA* models. ^b^ Standard errors estimated by jack-knife are approximate and particularly inaccurate for seasons because surveys in territories lasted 40 days, but some foxes associated in the same season but in different territories or years.

**Table D. Mantel test correlations (*R*) between seasonal association matrices with the mean *p*-value and standard deviation (SD) calculated from three runs each with 10,000 permutations.** Season pair denotes the two surveys compared, listed in the order of data collection: spring (SP), summer (SU), autumn (AU) and winter (WI). Time between seasons indicates how close in time the compared surveys were: 1 = consecutive, 2 = gap of one season and 3 = gap of 2 seasons. Significant *p*-values (*p* < 0.05) and those close to significance (0.1 > *p* > 0.05, marked with *) are shown in bold.

| **Territory** | **Season pair** | **Time between**  **seasons** | ***N* foxes** |  |  |  | **Mantel z-test** | |  | |  |  |  |  | **Dietz *R* test** | |  | |
| --- | --- | --- | --- | --- | --- | --- | --- | --- | --- | --- | --- | --- | --- | --- | --- | --- | --- | --- |
|  |  |  |  |  |  |  | ***R*** | **Mean *p*-value** | **SD** |  |  |  |  |  | **Dietz*-R*** | **Mean *p*-value** | **SD** |  |
| T1 | SU-AU | 1 | 6 |  |  |  | 0.957 | **0.002** | <0.001 |  |  |  |  |  | 0.959 | **0.001** | <0.001 |  |
|  | AU-WI | 1 | 11 |  |  |  | 0.280 | **0.098*** | 0.002 |  |  |  |  |  | 0.425 | **0.026** | 0.001 |  |
|  | WI-SP | 1 | 13 |  |  |  | 0.453 | **0.012** | <0.001 |  |  |  |  |  | 0.635 | **<0.001** | <0.001 |  |
|  | AU-SP | 2 | 11 |  |  |  | 0.169 | 0.165 | 0.001 |  |  |  |  |  | 0.529 | **0.003** | <0.001 |  |
|  | SU-WI | 2 | 6 |  |  |  | -0.015 | 0.448 | 0.008 |  |  |  |  |  | 0.293 | 0.165 | 0.122 |  |
|  | SU-SP | 3 | 6 |  |  |  | 0.303 | **0.059*** | 0.001 |  |  |  |  |  | 0.408 | **0.056*** | 0.004 |  |
| T2 | AU-WI | 1 | 5 |  |  |  | 0.861 | **0.016*** | 0.001 |  |  |  |  |  | 0.665 | **0.016** | <0.001 |  |
|  | WI-SP | 1 | 5 |  |  |  | 0.274 | 0.399 | 0.004 |  |  |  |  |  | -0.049 | 0.542 | 0.003 |  |
|  | SP-SU | 1 | 5 |  |  |  | -0.116 | 0.736 | 0.006 |  |  |  |  |  | -0.262 | 0.761 | 0.004 |  |
|  | AU-SP | 2 | 5 |  |  |  | 0.546 | 0.182 | 0.003 |  |  |  |  |  | 0.622 | 0.177 | 0.001 |  |
|  | WI-SU | 2 | 5 |  |  |  | 0.571 | **0.027** | 0.001 |  |  |  |  |  | 0.535 | **0.066*** | 0.002 |  |
|  | AU-SU | 3 | 5 |  |  |  | 0.450 | 0.143 | 0.004 |  |  |  |  |  | 0.233 | 0.287 | 0.002 |  |
| T3 | AU-WI | 1 | 6 |  |  |  | 0.960 | **0.006** | <0.001 |  |  |  |  |  | 0.878 | **0.023** | 0.001 |  |
|  | WI-SP | 1 | 7 |  |  |  | 0.804 | **0.017** | 0.001 |  |  |  |  |  | 0.748 | **0.019** | 0.001 |  |
|  | SP-SU | 1 | 5 |  |  |  | 0.978 | **0.009** | 0.001 |  |  |  |  |  | 0.957 | **0.008** | 0.001 |  |
|  | AU-SP | 2 | 6 |  |  |  | 0.640 | **0.076*** | 0.003 |  |  |  |  |  | 0.690 | **0.061*** | 0.003 |  |
|  | WI-SU | 2 | 5 |  |  |  | 0.701 | 0.115 | 0.002 |  |  |  |  |  | 0.678 | **0.067*** | 0.001 |  |
|  | AU-SU | 3 | 5 |  |  |  | 0.511 | 0.184 | 0.001 |  |  |  |  |  | 0.436 | 0.220 | 0.005 |  |
| T4 | AU-WI | 1 | 6 |  |  |  | 0.495 | 0.100 | 0.002 |  |  |  |  |  | 0.660 | **0.058*** | 0.002 |  |
|  | WI-SP | 1 | 4 |  |  |  | -0.463 | 0.748 | 0.006 |  |  |  |  |  | -0.224 | 0.668 | 0.002 |  |
|  | SP-SU | 1 | 4 |  |  |  | 0.089 | 0.460 | 0.002 |  |  |  |  |  | 0.221 | 0.293 | 0.003 |  |
|  | AU-SP | 2 | 4 |  |  |  | 0.676 | 0.123 | 0.002 |  |  |  |  |  | 0.928 | **0.082*** | 0.003 |  |
|  | WI-SU | 2 | 4 |  |  |  | -0.050 | 0.623 | 0.003 |  |  |  |  |  | 0.000 | 0.584 | 0.007 |  |
|  | AU-SU | 3 | 4 |  |  |  | 0.306 | 0.167 | 0.004 |  |  |  |  |  | 0.319 | 0.211 | 0.003 |  |
| T5 | WI-SP | 1 | 7 |  |  |  | 0.838 | **0.001** | <0.001 |  |  |  |  |  | 0.799 | **0.005** | <0.001 |  |
|  | SP-SU | 1 | 6 |  |  |  | 0.552 | **0.068*** | 0.001 |  |  |  |  |  | 0.329 | 0.164 | 0.001 |  |
|  | SU-AU | 1 | 4 |  |  |  | 0.827 | **0.085*** | 0.001 |  |  |  |  |  | 0.938 | **0.082*** | 0.004 |  |
|  | SP-AU | 2 | 4 |  |  |  | 0.783 | 0.125 | 0.004 |  |  |  |  |  | 0.281 | 0.335 | 0.003 |  |
|  | WI-SU | 2 | 6 |  |  |  | 0.541 | **0.063*** | 0.003 |  |  |  |  |  | 0.288 | 0.225 | 0.003 |  |
|  | WI-AU | 3 | 4 |  |  |  | 0.854 | **0.082*** | 0.006 |  |  |  |  |  | 0.563 | 0.127 | 0.001 |  |
| T6 | SU-AU | 1 | 9 |  |  |  | 0.494 | **0.012** | 0.001 |  |  |  |  |  | 0.618 | **0.001** | <0.001 |  |
|  | AU-WI | 1 | 14 |  |  |  | 0.506 | **0.004** | <0.001 |  |  |  |  |  | 0.534 | **<0.001** | <0.001 |  |
|  | WI-SP | 1 | 6 |  |  |  | 0.448 | **0.086*** | 0.003 |  |  |  |  |  | 0.387 | 0.139 | 0.001 |  |
|  | AU-SP | 2 | 4 |  |  |  | 0.612 | 0.248 | 0.005 |  |  |  |  |  | 0.885 | 0.167 | 0.006 |  |
|  | SU-WI | 2 | 10 |  |  |  | 0.253 | **0.095*** | 0.003 |  |  |  |  |  | 0.383 | **0.037** | 0.001 |  |
|  | SU-SP | 3 | 3 |  |  |  | 0.847 | 0.331 | 0.001 |  |  |  |  |  | 0.500 | 0.502 | 0.001 |  |
| T7 | SU-AU | 1 | 4 |  |  |  | 0.865 | **0.084*** | 0.004 |  |  |  |  |  | 0.893 | 0.125 | 0.001 |  |
|  | AU-WI | 1 | 5 |  |  |  | 0.886 | **0.042** | 0.002 |  |  |  |  |  | 0.871 | **0.060*** | 0.003 |  |
|  | WI-SP | 1 | 4 |  |  |  | 0.080 | 0.503 | 0.005 |  |  |  |  |  | -0.143 | 0.751 | 0.003 |  |
|  | AU-SP | 2 | 4 |  |  |  | 0.493 | 0.207 | 0.005 |  |  |  |  |  | 0.314 | 0.329 | 0.006 |  |
|  | SU-WI | 2 | 4 |  |  |  | 0.941 | **0.041** | 0.001 |  |  |  |  |  | 0.955 | **0.040** | 0.003 |  |
|  | SU-SP | 3 | 3 |  |  |  | 0.612 | 0.331 | 0.004 |  |  |  |  |  | 0.500 | 0.500 | 0.009 |  |
